# Water-soluble fluorinated copolymers as highly sensitive ^19^F MRI tracers: From structure optimization to multimodal tumor imaging

**DOI:** 10.1016/j.mtbio.2025.101462

**Published:** 2025-01-04

**Authors:** Tuba Ayça Tunca Arın, Dominik Havlíček, Diego Fernando Dorado Daza, Natalia Jirát-Ziółkowska, Ognen Pop-Georgievski, Daniel Jirák, Ondrej Sedlacek

**Affiliations:** a Department of Physical and Macromolecular Chemistry, Faculty of Science, Charles University, Prague 2, 128 00, Czech Republic; b Department of Diagnostic and Interventional Radiology, Institute for Clinical and Experimental Medicine, Prague 4, 140 21, Czech Republic; c Institute of Biophysics and Informatics, First Faculty of Medicine, Charles University, Prague 2, Prague, 128 00, Czech Republic; d Department of Chemistry and Physics of Surfaces and Interfaces, Institute of Macromolecular Chemistry, AS CR, Prague 6, 162 06, Czech Republic; e Faculty of Health Studies, Technical University of Liberec, Liberec, 461 17, Czech Republic

## Abstract

Fluorine magnetic resonance imaging (^19^F MRI) using polymer tracers overcomes limitations of conventional proton MRI by offering enhanced specificity. However, the lack of systematic comparisons among fluorinated polymers has hindered rational tracer design. In this study, we synthesized an extensive library of water-soluble fluorinated copolymers by varying ratios of hydrophilic and fluorinated monomers and evaluated their ^19^F MRI properties to identify key structure–property relationships. Optimizing the hydrophilicity of the non-fluorinated comonomer increased fluorine content without compromising water solubility, thereby enhancing the MRI signal. Factors such as chemical structure, molecular interactions, and magnetic relaxation times also significantly influenced tracer performance. The optimized copolymer, poly((*N*-(2,2,2-trifluoroethyl)acrylamide)_60_-*stat*-(*N*-(2-hydroxyethyl)acrylamide)_40_), exhibited unprecedented ^19^F MRI sensitivity with detection limits below 1 mg mL^−1^, the highest reported to date. We demonstrated the tracer's potential through successful *in vivo*^19^F MRI visualization of solid tumors in mouse models, highlighting its promise for advanced biomedical imaging applications.

## Graphical abstract

**Figure:**
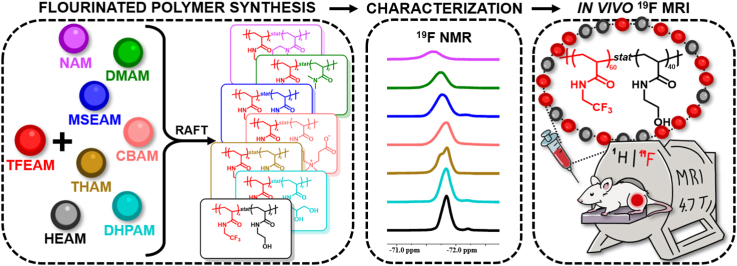

## 1 Introduction

Conventional hydrogen (^1^H) magnetic resonance imaging (MRI) is a state-of-the-art, non-invasive medical imaging technology that provides detailed insights into internal structures of the human body without requiring ionizing radiation [1,2]. However, ^1^H MRI suffers from widespread background signals from water and carbohydrates and ensuing problems [3,4]. In response to this challenge, researchers have developed an alternative approach, more specifically fluorine (^19^F) MRI [[5], [6], [7]].

^19^F MRI may be characterized as “hotspot imaging” because, unlike hydrogen, only trace amounts of fluorine are found in living organisms, facilitating the visualization of fluorinated molecules (tracers) by ^19^F MRI without physiological background signals [8]. Nevertheless, several other factors explain why fluorine is such an excellent choice for MRI. Case in point, fluorine has 100 % natural abundance and a gyromagnetic ratio close to that of hydrogen, so only minor hardware modifications of clinical instruments are needed to efficiently visualize fluorinated tracers [9,10].

Among the most widely studied fluorinated tracers, perfluorocarbons (PFCs) stand out for their relatively high fluorine content and sensitivity [[11], [12], [13]]. Notwithstanding these properties, they are extremely hydrophobic and, hence, poorly soluble in water. To prevent their aggregation or precipitation, PFCs must be encapsulated into nanoscale systems, such as micelles [14,15]. Therefore, currently available fluorine tracers limit the potential of ^19^F MRI as their widespread use in the medical field is prevented by the need for encapsulation and by their inherent hydrophobicity.

Water-soluble fluorinated polymers have recently emerged, opening up promising avenues for enhanced imaging [16,17]. However, this new class of ^19^F MRI tracers remains incipient due to the inherent hydrophobicity of fluorine atoms, which adversely affects polymer solubility in water. Preparing water-soluble fluorinated polymers with good MR relaxation requires optimizing their fluorine content [18,19]. So far, water-soluble fluorinated polymers have been prepared using two synthetic strategies.

Homopolymerization of hydrophilic semifluorinated monomers, such as *N*-(2-((2,2,2-trifluoroethyl)sulfinyl)ethyl)acrylamide (FSAM) [20], *N*-(2-fluoroethyl)acrylamide) [21] and semifluorinated zwitterions [[22], [23], [24]], leads to well-defined hydrophilic fluorinated polymers, albeit constrained by oft-challenging monomer synthesis and by limited options for fluorine content optimization. By contrast, the synthesis of statistical copolymers of hydrophobic fluorinated monomers (e.g., *N*-(2,2,2-trifluoroethyl)acrylamide) with hydrophilic non-fluorinated monomers (e.g., *N*-(2-hydroxyethyl)acrylamide)) provides a straightforward route toward hydrophilic fluorinated polymers. Moreover, both their fluorine content and water solubility can be easily adjusted by changing the chemical structure and ratio of both comonomers in the copolymer, as shown by several studies [16].

Recent research has highlighted the potential of such copolymeric systems in ^19^F MRI applications. For example, Whittaker and colleagues have prepared excellent ^19^F MRI probes from several copolymers of hydrophilic 2-(methylsulfinyl)ethyl acrylate and hydrophobic fluorinated 2,2,2-trifluoroethyl acrylate with exceptional imaging performance both *in vitro* and *in vivo* [25]. Similarly, from three different hydrophobic fluorinated monomers, Gao and colleagues designed pH-responsive copolymers with tertiary amine-containing repeating units, yielding water-soluble probes at a pH below the p*K*_a_ for a multi-chromatic pH-activatable system [26]. Yet, despite the number of studies on MRI tracers based on fluorinated polymers, to the best of our knowledge, no report has ever compared tracers with different structures to correlate polymer structure with ^19^F MR properties. This knowledge gap precludes a systematic approach to tracer design.

This article aims to assess how the structure of water-soluble semifluorinated copolymers affects their ^19^F MR properties, providing guidelines for the rational design of highly sensitive tracers for advanced imaging. To this end, a comprehensive library of different water-soluble semifluorinated copolymers based on substituted polyacrylates and polyacrylamides was synthesized using a wide range of hydrophilic monomers in combination with various fluorinated comonomers. Copolymers were synthesized with an increasing content of the fluorinated repeating unit (RU) up to the threshold of water-soluble copolymers. All copolymers were compared based on their ^19^F MR properties, thereby identifying the key parameters that lead to superior MR performance. The optimal copolymer, poly((*N*-(2,2,2-trifluoroethyl)acrylamide)_60_-*stat*-*(N*-(2-hydroxyethyl)acrylamide)_40_), showed excellent ^19^F MRI sensitivity and no cytotoxicity. Furthermore, this copolymer was successfully visualized *in vivo* by ^19^F MRI in tumor-bearing mice, demonstrating the ability to detect tumor tissue by ^19^F MRI. These findings underscore the potential of these copolymers for advanced imaging applications.

## 2 Experimental section

### 2.1 Materials

*N*-(2,2,2-Trifluoroethyl)acrylamide (TFEAM) [27], 2-(methylsulfinyl)ethyl acrylamide (MSEAM) [28], 2-(*n*-butyltrithiocarbonate) propionic acid (BTPA) [29], methyl 2-(*n*-butyltrithiocarbonate) propanoate (MBTPA) [30] and *N*-boc-aminoethyl acrylamide (Boc-AEAM) [31] were synthesized as reported in the literature. In addition, *N*-2,2,2-Trifluoroethylamine was purchased from TCI; Sulfo-Cyanine 7 NHS ester (Cy7), from Lumiprobe; and all other chemicals, from Sigma-Aldrich, unless stated otherwise. Liquid monomers (*N*-hydroxyethyl acrylamide (HEAM), *N*-hydroxyethyl acrylate (HEA), *N*,*N*-dimethylacrylamide (DMAM), and *N*-(2,2,2-trifluoroethyl)acrylate (TFEA)) were filtered through a short pad of basic alumina before use to remove the inhibitor. Calculated logarithm of the partition function (cLogP) values of monomers were calculated by ChemDraw. Water was deionized with a Millipore Milli-Q water purification system.

### 2.2 Synthesis of monomers and copolymers

#### 2.2.1 Synthesis of *N*-(2,3-dihydroxypropyl)acrylamide (DHPAM)

(±)-3-Amino-1,2-propanediol (5 g, 50.4 mmol) and triethylamine (TEA, 14.1 mL, 101 mmol) were dissolved in the methanol and acetonitrile (1:1, 200 mL) mixture and cooled in an ice bath. Acryloyl chloride (4.10 mL, 50.4 mmol) in acetonitrile (20 mL) was added dropwise, and the reaction was stirred at room temperature overnight. Subsequently, the insoluble salts were filtered off, and the crude mixture was evaporated under reduced pressure. Lastly, the product was purified by column chromatography on silica using a chloroform-methanol (9:1) mobile phase. After evaporation, DHPAM was obtained in 47 % yield as a pale yellow oil that crystallized upon freezing. ^1^H NMR (DMSO-*d*_6_): δ 8.41 (bs, 1H), 6.16–6.26 (dd, 1H), 6.04–6.14 (dd, 1H), 5.56–5.64 (dd, 1H), 3.59–3.48 (ddt, 1H), 3.48–3.37 (ddt, 1H), 2.90–3.01 (ddd, 1H), 2.74–2.84 (dt, 1H), 2.57 (s, 3H).

#### 2.2.2 Synthesis of copolymers

Semifluorinated copolymers were synthesized by statistical RAFT copolymerization of a fluorinated monomer and a hydrophilic comonomer. All polymerizations were conducted at 70 °C under a nitrogen atmosphere, with a target degree of polymerization (DP) of 100, using 2-(*n*-butyltrithiocarbonate) propionic acid (BTPA) as the chain transfer agent (CTA) and azobisisobutyronitrile (AIBN) as the initiator. In a typical RAFT polymerization procedure for P1_60_, TFEAM (100 mg, 0.65 mmol), HEAM (50 mg, 0.44 mmol), BTPA (2.6 mg, 10 μmol) and AIBN (0.6 mg, 3.6 μmol) were dissolved in DMF (1 mL) and purged with nitrogen gas. The reaction was stirred at 70 °C for 4 h. For 3-((3-acrylamidopropyl)dimethylammonio)propanoate (carboxybetaine acrylamide, CBAM) and *n*-[tris(hydroxymethyl)methyl]acrylamide (THAM) copolymers, 4,4′-azobis(4-cyanovaleric acid) (ACVA) was used as the initiator, and a mixture of equal volumes of DMF and water was used as the copolymerization solvent. The concentrated crude copolymers were purified by gel filtration on a PD-10 column, in water, followed by freeze-drying.

#### 2.2.3 Synthesis of the fluorescently labeled copolymer for the *in vivo* study

For the *in vivo* study, the TFEAM-*stat*-HEAM (P1) copolymer was prepared with a target DP of 300 for enhanced permeation and retention (EPR) effect and *N*-Boc-aminoethyl acrylamide (Boc-AEAM) was added for fluorescent dye conjugation. To this end, TFEAM (504 mg, 3.3 mmol), HEAM (270 mg, 2.3 mmol), Boc-AEAM (50 mg, 0.23 mmol), MBTPA (5 mg, 19.8 μmol) and AIBN (0.6 mg, 3.6 μmol) were dissolved in DMF (3 mL). After purging with nitrogen gas, the reaction was stirred at 70 °C for 16 h. To remove the protecting group, the copolymer (350 mg) was dissolved and stirred in methanolic HCl (2.3 M, 3.5 mL) overnight and then precipitated into diethyl ether and dried. The resulting amine-containing copolymer (350 mg), sulfo-Cyanine 7 NHS ester (7 mg, 8.2 μmol) and TEA (12 μL, 86.1 μmol) were dissolved in DMF (1 mL) and stirred overnight at room temperature followed by acetic anhydride (4 μL, 42.4 μmol) addition to block potentially unreacted primary amines. The product was purified by gel filtration on a Sephadex LH-20 column using methanol as the eluent, evaporated under reduced pressure, dissolved in distilled water and freeze-dried.

### 2.3 Polymer characterization

*Size exclusion chromatography* (SEC) was performed to determine the molecular weights (*M*_w_ - mass-averaged molecular weight, *M*_n_ - number-averaged molecular weight) and dispersity (*Ð* = *M*_w_/*M*_n_) of the polymers on a Malvern OMNISEC or a Watrex Streamline system. Malvern OMNISEC system was equipped with an autosampler, a two-angle light scattering detector (right-angle light scattering (RALS): 90° angle; low-angle light scattering (LALS): 7° angle), a differential refractive index detector, a viscometer and a diode-array-based UV/Vis spectrometer. The separation was performed on two PLgel 5 μm, 7.5 × 300 mm mixed-D columns in series thermostatted at 55 °C in *N,N*-dimethylacetamide (DMAc) containing 50 mM of LiCl at 0.5 mL min^−1^ elution rate. Molar masses and dispersities were calculated against narrow-dispersity poly(methyl methacrylate) standards. Carboxybetaine-containing copolymers were characterized using a Watrex Streamline system equipped with a Streamline P1 Pump, a Streamline AS2 Autosampler, a Streamline CT Column Thermostat, a Streamline UV detector and a Streamline RI detector. The separation was performed on a NOVEMA Max 100 Å, 8 × 300 mm, 5 μm SEC column thermostatted at 50 °C using a methanol and sodium acetate buffer (pH 5.45) mixture (80:20 v/v) as the mobile phase at 0.5 mL min^−1^ elution rate. Molar masses and dispersities were calculated against dextran standards.

*Nuclear magnetic resonance* (NMR) spectra were recorded on a Bruker Advance MSL 400 MHz NMR spectrometer at 25 °C in DMSO-*d*_6_, D_2_O or a H_2_O/D_2_O (95/5 v/v) mixture. Unless stated otherwise, all ^19^F NMR spectra were measured at *c*_pol_ = 10 mg mL^−1^ with trifluoroethanol standard using 20 μs pulse width, 8 s relaxation delay and 1.5 s acquisition time over 64 scans, expressing all chemical shifts as ppm. The NMR spectra were processed in MestReNova 14.1 software, and the signal-to-noise ratios were calculated using the built-in MestReNova function.

*Dynamic light scattering* (DLS) measurements were performed to determine the hydrodynamic diameters of the polymers in distilled water on a ZEN3600 Zetasizer Nano-ZS zeta potential analyzer (Malvern Instruments, UK). The polymer samples (*c*_pol_ = 5 or 10 mg mL^−1^) were filtered through a 0.22-μm polytetrafluoroethylene (PTFE) syringe filter before each measurement. The apparent volume-weighted hydrodynamic diameter of the copolymers, *D*_h_, was determined at *θ* = 173° scattering angle, using the DTS (Nano) program to analyze the data.

### 2.4 Magnetic resonance relaxometry

The ^1^H and ^19^F MR relaxation times of the copolymers were determined using a 1.5 T Minispec 60 MHz relaxometer (Bruker Biospin, Germany) with interchangeable radiofrequency (RF) coils tuned to an optimal resonance frequency of 60 MHz for ^1^H and 54 MHz for ^19^F, respectively. The *T*_1_ relaxation times were measured using an inversion recovery sequence with the following parameters: repetition time, TR = 0.01–10,000 ms; recycle delay = 1 s; scans = 4; 10 points per fitting. The *T*_2_ relaxation times were measured using the Carr-Purcell-Meiboom-Gill (CPMG) sequence: echo time, TE = 0.04 ms; TR = 5000 ms; recycle delay = 2 s; number of scans = 8; 20,000 points per fitting.

### 2.5 Magnetic resonance imaging and spectroscopy

MR properties of fluorinated copolymer tracers were measured on a 4.7 T spectrometer (Bruker Biospec 47/20, Ettlingen, Germany). For *in vitro* phantom characterization, a volume birdcage RF coil was selected because this coil is optimal for measuring multiple phantoms due to its homogeneity [32], whereas *in vivo* measurements were performed using a surface coil optimized for mice experiments. Both RF coils were double-tuned and matched to the Larmor frequencies of both ^1^H and ^19^F nuclei for optimal performance without changing the coil during the experiment. Reference hydrogen (^1^H) MR images were acquired using a *T*_2_-weighted fast spin-echo sequence with the following parameters: TR = 3300 ms; TE = 11.70 ms; effective echo time, TE_eff_ = 35.10 ms; spatial resolution = 0.156 × 0.156 mm^2^; number of acquisitions, NA = 1; turbo factor = 8; scan time = 1 min 19 s.

To characterize the fluoropolymers, tracer MR sensitivity was analyzed by ^19^F MR spectroscopy (^19^F MRS) using a single-pulse sequence (TR = 500 ms; number of averages = 2–600) and by ^19^F MRS imaging (^19^F MRSI), a spectrally resolved method that is used to acquire ^19^F MR images from a 2D slab of voxels with the following parameters: TR = 200 ms; TE = 15 ms; bandwidth, BW = 38 ppm; spatial resolution = 2.5 × 2.5 × 20 mm^3^, scan time = 1–120 min. *In vivo* ^19^F MR images were also taken using the MRSI method, with the same imaging parameters, albeit setting the scan time to 30 min.

### 2.6 Magnetic resonance data processing and quantitation

^19^F MRS and ^19^F MRSI data processing and quantification were primarily performed in the MATLAB programming environment (Matlab R2022a, The MathWorks, Inc., USA) using custom-written scripts. For non-localized spectroscopy, we assessed tracer sensitivity by calculating the signal-to-noise ratio (*SNR*_MRS_) as the ratio of the signal amplitude (*Sa*) to the standard deviation of noise (*Nσ)* (Equation (1)).

(1) $${\text{SNR}}_{\mathrm{MRS}=}\frac{S_{a}}{N_{\sigma }}$$

For ^19^F hotspot MRSI, the resulting phantom images were reconstructed as normalized maximum intensity projections (MIP) from 40 slices, corresponding to approximately 3 ppm. We employed bilinear interpolation to transform the images from a 64 × 64 matrix to a 256 × 256 matrix, aligning them with the dimensions of the reference ^1^H image. The SNR of ^19^F MRSI (SNR_MRSI_) images was calculated by dividing the mean signal (*S*) in a region of interest (ROI) by the average standard deviation of noise (*σ*) at the corners of the image. To address the inherent Rician noise distribution [33] in MR images, a correction factor of 0.655 was included in the SNR calculation (Equation (2)).

(2) $${\text{SNR}}_{\mathrm{MRSI}=0.655∙}\frac{S}{\sigma }$$

### 2.7 Cytotoxicity

Polymer cytotoxicity was tested using a murine mammary carcinoma 4T1 cell line, which is suitable for preliminary tests prior to *in vivo* experiments. The AlamarBlue Cell Viability Reagent (Thermo Fisher Scientific, USA) is a resazurin-based solution that quantitatively measures cell viability. First, 4T1 cells were cultured in RPMI medium supplemented with 10 % fetal bovine serum, 0.5 % penicillin/streptomycin and 4 mmol L^−1^ L-glutamine (Thermo Fisher Scientific, USA) under standard conditions (5 % CO_2_, 37 °C). The cells were then counted on a Vi-CELL XR Cell Viability Analyzer (Beckman Coulter, USA), seeded in 96-well plates (1 × 10^4^ cells per well) and incubated for 24 h. Once the cells reached 70 % confluence, the polymer was diluted in fresh growth medium to final concentrations of *c*_pol_ = 10, 15 and 20 mg mL^−1^ and added to the wells in triplicates (100 μL per well). The control cells received fresh growth medium without the polymer. The contrast agent effect on cell viability was assessed after 24 h of incubation, followed by 4 h of incubation with 10 % AlamarBlue. Resazurin, the active component of the reagent, is visibly reduced to resorufin only in viable cells, indicating their metabolic activity. Absorbance in test and control wells was measured at 570/600 nm on an Elisa Microplate Reader RT-6900 (Gen5 Software, BioTek, Germany). The results were quantified as a percentage of resazurin reduction. The assay was repeated three times.

### 2.8 Hemolysis assay

Blood compatibility of the polymer was tested by the hemolysis assay. Human blood was collected from a healthy volunteer into heparin-coated vacutainers (Becton Dickinson Czechia Ltd., Prague, Czech Republic). The plasma was removed by centrifugation at 3000 rpm for 10 min, followed by the washing of red blood cells (RBCs) three times with cold PBS (Phosphate buffered saline tablets, Sigma Aldrich). Then, the suspension was diluted with PBS to the final concentration matching the full blood dilution of 1/49.

For the hemolysis test, several dilutions of P1_60_ (from 10 mg mL^−1^ to 0.156 mg mL^−1^) were prepared in triplicates, and deionized water and PBS were used as the positive and negative controls, respectively. 1 mL of each was mixed with 0.3 mL of diluted RBCs and incubated at 37 °C for 6 or 12 h. After all the samples were centrifuged at 3000 rpm for 10 min, their absorbance values were determined by UV–vis spectrometry at 541 nm. The percent hemolysis was calculated by the following equation (3):

(3) $$Hemolysis\;\%=\frac{Absorbance\;sample-Negative\;control\;}{Positive\;control-Negative\;control}\times 100$$

### 2.9 Fluorescence imaging and quantitation

An AMI HT optical imaging system (Spectral instruments imaging, USA) was used for both *in vivo* and *ex vivo* fluorescence imaging of the biodistribution of the P1_60_-Cy7 tracer. The Cy7 fluorescence dye has an excitation peak at 750 nm and an emission peak at 773 nm, so the measurements were performed using the following parameters: Excitation filter = 745 nm; Emission filter = 790 nm; Exposure time = 2 s; Field of View (FOV) = 15 cm, Binning = 2. Both *in vivo* and *ex vivo* data were quantified using the software analysis tool Aura (Spectral Instruments Imaging, USA). The appropriate ROIs surrounding the tissues were selected with the Freehand tool to assess the total emission (photons/second).

### 2.10 *In vivo* distribution of P1_60_ in 4T1 cells-induced tumor-bearing mice

The *in vivo* distribution of P1_60_ was analyzed in female BALB/c mice, including tumor-bearing mice (n = 3) and a negative control mouse without a tumor. For the tumor-bearing group, tumorous 4T1 cells (1 × 10⁶ cells in 100 μL PBS per mouse) were subcutaneously injected into the right flank, approximately midway between the lower rib cage and the hind limb, avoiding major blood vessels and nerves. The tumors were allowed to grow for 19 days prior to further MR and optical imaging measurements. The negative control mouse was included to assess the distribution of the tracer in the absence of a tumor. To improve the sensitivity of fluorescence imaging, the chest and abdomen were shaved to suppress incoming and outgoing light absorption and scattering. Subsequently, the PBS solution of P1_60_, conjugated with the Cy7 dye, was systematically injected via the retro-orbital sinus (*V* = 100 μL, *c*_pol_ = 70 mg mL^−1^), which is a relatively easy and reliable pathway for intravascular delivery. Measurements were performed at five post-injection (PI) time points (15 min and 1, 4, 24, and 48 h) for fluorescence imaging and at three time points (4, 24, and 48 h) for MR measurements. General anesthesia was induced with 5 % isoflurane gas (Baxter, Deerfield, USA), maintained at 1.5–0.5 % during imaging. Because the MRI measurements were longer than fluorescence imaging, additional precautions were taken, including applying an eye gel (Ophtalmo-Septonex, Zentiva, Czech Republic) to prevent corneal damage and monitoring the respiratory rate using a pressure sensor (Rapid Biomedical, Berlin, Germany) positioned beneath the abdomen. After completing all *in vivo* measurements, the animals were sacrificed with an anesthetic gas overdose for vital organ extraction. The organs were used for *ex vivo* analysis. All animal protocols were approved by the Ethics Committee of the Institute for Clinical and Experimental Medicine and the Ministry of Health of the Czech Republic (no. 58/2014) in accordance with the European Communities Council Directive (2010/63/EU).

## 3 Results and discussion

### 3.1 Synthesis of semifluorinated copolymers

To assess the impact of polymer structure on ^19^F MRI performance, an extensive library of water-soluble fluorinated copolymers varying in comonomer structures and ratios were synthesized by reversible addition-fragmentation chain transfer (RAFT) copolymerization of fluorinated and hydrophilic comonomers with a target DP of 100, to obtain nearly random architectures. Copolymers were prepared with different comonomer ratios because their water solubility depends on fluorine content and monomer hydrophilicity. The fluoromonomer content was increased in each series until the copolymer remained water soluble at room temperature (*c*_pol_ = 10 mg mL^−1^). All copolymers were characterized by ^1^H NMR spectroscopy to ensure that the copolymer composition matched the comonomer feed ratio (Figs. S1–10). Size-exclusion chromatography (SEC) analysis confirmed the expected molar mass and low dispersity of the copolymers.

### 3.2 Comparison of semifluorinated polyacrylamides and polyacrylates

**Fig. 1:**
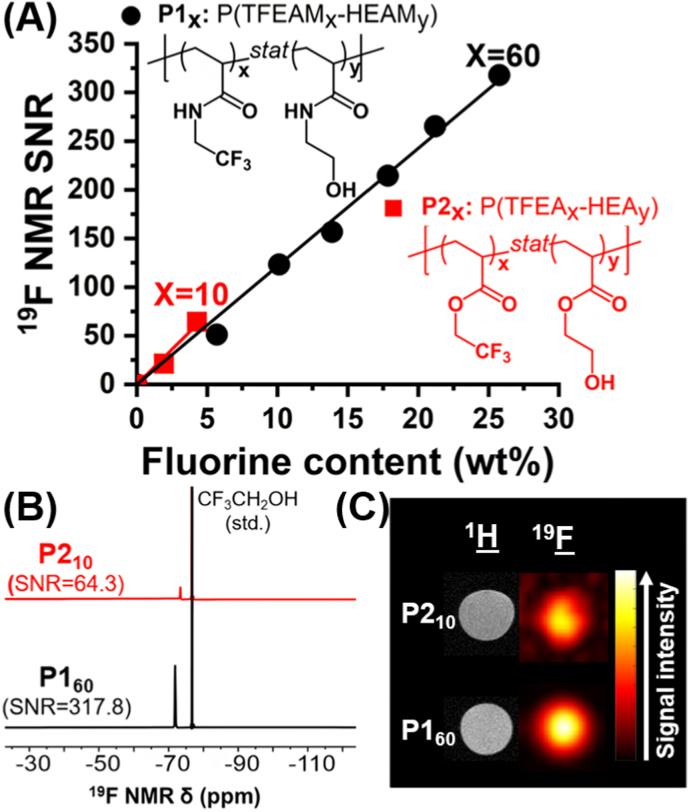
Water-soluble semifluorinated acrylamide copolymers (P1_x_) outperform polyacrylate analogs (P2_x_) in fluorine content and ^19^F MRI properties. (A) Variation of ^19^F NMR SNR as a function of fluorine content. (B) Representative ^19^F NMR spectra of water-soluble acrylamide and polyacrylate copolymers with the highest fluorine content, namely P1_60_ (P(TFEAM_60_-HEAM_40_)) and P2_10_ (P(TFEA_10_-HEA_90_)), respectively. (C) ^1^H and ^19^F MRSI of these copolymers in water at *c*_pol_ = 10 mg mL^−1^ and 30-min acquisition time.

To directly compare ^19^F MR properties of acrylamide and acrylate-based copolymers, TFEAM-*stat*-HEAM (P1, representative fluorinated polyacrylamides) and TFEA-*stat*-HEA (P2, representative polyacrylates) counterparts were synthesized in varying comonomer ratios (Fig. 1). The molar content of each fluorinated comonomer is shown in subscript (e.g., P1_60_ stands for P(TFEAM_60_-*stat*-HEAM_40_)). Polyacrylamides, being more hydrophilic than polyacrylates, allowed water-soluble copolymers to achieve significantly higher fluorine contents. Specifically, acrylamide-based copolymers peaked at 25 wt% fluorine (P1_60_, with 60 mol% TFEAM), whereas polyacrylate-based analogs reached only 4 wt% fluorine (P2_10_, corresponding to 10 mol% TFEA). This significant difference in fluorine content between series was also assessed by ^19^F NMR, with P1_60_ showing a much higher signal-to-noise ratio (SNR) in water (317.8) than P2_10_ (64.3) at the same copolymer concentration (*c*_pol_ = 10 mg mL^−1^, Fig. 1B). A similar trend was observed *in vitro* by ^19^F MRI in Eppendorf tube phantoms, where P1_60_ (SNR = 139.0) significantly outperformed P2_10_ (SNR = 18.7) (Fig. 1C). Therefore, semifluorinated acrylamide copolymers were selected for further tests.

### 3.3 Hydrophilic comonomer effects on ^19^F MR properties

**Fig. 2:**
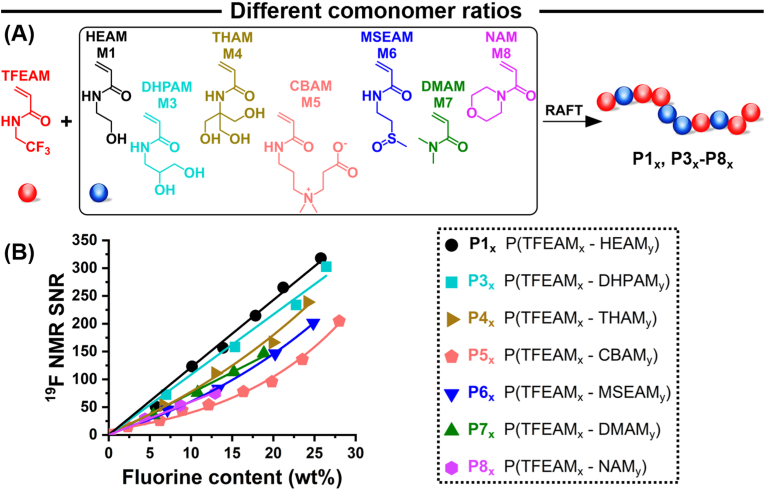
The ^19^F NMR (400 MHz) signal-to-noise ratio of semifluorinated copolyacrylamides increases with the fluorine content of their hydrophilic comonomer. (A) Schematic representation of statistical copolymerization of TFEAM with various hydrophilic comonomers. (B) Variation of ^19^F NMR SNR as a function of fluorine weight percent of all copolymer compositions, where x = TFEAM DP, and y = hydrophilic comonomer DP, with a total DP of 100.

We evaluated the impact of different hydrophilic comonomers on ^19^F MR signal intensity using a library of water-soluble semifluorinated polyacrylamides. Various hydrophilic acrylamide monomers, each with unique structural features and hydrophilicities, were copolymerized with TFEAM at different ratios (Fig. 2A). In this study, we optimized tracer sensitivity at the lowest observable polymer concentration, as the total injected polymer amount is often the limiting factor in ^19^F MRI due to the inherently lower sensitivity of this method. While one might achieve higher sensitivity in terms of fluorine concentration ([F]) by incorporating just a tiny amount of fluorinated monomer into a highly hydrophilic polymer (due to superior relaxation), this strategy would require injecting large total quantities of polymer to deliver sufficient fluorine for imaging. Such high polymer doses could raise concerns regarding potential toxicity and unfavorable biodistribution. Therefore, our approach focuses on maximizing the fluorine content within a water-soluble copolymer to enhance ^19^F MRI sensitivity while minimizing the total polymer dose.

Hydroxylated monomers HEAM (M1), DHPAM (M3), and THAM (M4) contain one, two, and three hydroxyl groups, respectively. In turn, the CBAM monomer (M5) is a zwitterion, and the MSEAM monomer (M6) contains a polar sulfoxide moiety. Lastly, DMAM (M7) and NAM (M8) are hydrophilic monomers widely used in biomedical applications but are poorly hydrophilic due to their tertiary amides with low hydrogen bonding potential. Water-soluble copolymers (*c*_pol_ = 10 mg mL^−1^, for molar fluorine concentrations, see Tables S1–S10) of each type were prepared with an increasing TFEAM content.

All copolymers were studied by ^19^F NMR in water at the same polymer concentration (10 mg mL^−1^) to assess the effect of their structure on signal performance (Fig. 2B–S11). Hydroxyl-containing copolymers P1, P3, and P4 yielded the highest ^19^F NMR signal intensities. Conversely, tertiary amide-containing copolymers P7 and P8 had the lowest fluorine contents and NMR signal intensities. In all cases, ^19^F NMR SNR increased with the TFEAM content. This increase was linear across all copolymers with relatively short hydrophilic side-chains (P1, P3, P7, and P8), mirroring the theoretical relationship (Eq. (4)),

(4) $$I=\nu N\left(F\right)\left[1-2\;\exp\left(\frac{-\left(\text{TR}-\frac{\text{TE}}{2}\right)}{T_{1}}\right)+\exp\left(\frac{-\text{TR}}{T_{1}}\right)\right]\exp\left(\frac{-\text{TE}}{T_{2}}\right)$$

where *I* stands for MR signal intensity; *N*(F), for the number of ^19^F nuclei, which is proportional to fluorine content; *ν*, for the detectability of the fluorine nuclei; *T*_1_ and *T*_2_, for the ^19^F relaxation times; TE, for measurement echo time; and TR, for repetition time. In our study, the last two factors were close to unity as all copolymer *T*_1_ and *T*_2_ values (Fig. S12) were significantly higher than TR and TE values used for MR measurements, respectively.

In contrast to homopolymers, statistical copolymers are structurally irregular due to the varied combinations of neighboring repeating units in the polymer sequence. These variations lead to distinctive dyads and triads, which cause changes in NMR chemical shifts. Consequently, this leads to signal broadening and attenuation. The extent of chemical shift broadening is highly dependent on the structure of the hydrophilic comonomer. Notably, hydroxy-containing copolymers (P1) show a sharp singlet ^19^F NMR peak. Additionally, their signal intensity varies linearly with fluoromonomer content, indicating that the chemical shift of fluorinated units remains unchanged when PTFEAM units are replaced with PHEAM (Fig. S13).

This invariance may be explained by the similar steric and electronic features of TFEAM and HEAM side-chain groups, but TFEAM copolymers with bulky or strongly polar comonomers, namely P4 (THAM), P5 (CBAM), and P6 (MSEAM) (Fig. S13), show broader and non-uniform ^19^F NMR peaks. Such peaks indicate that the ^19^F chemical shift markedly varies with the type of neighboring repeating unit. Furthermore, the sequence irregularity effect is significantly stronger in copolymers with similar comonomer ratios than in those with very high or low fluoromonomer contents (where AA or BB-type homo-dyads prevail). Therefore, structural irregularity explains the non-linear variation of ^19^F NMR SNR as a function of fluoromonomer content for bulky and strongly polar unit-containing copolymers.

The increase in the slope of the ^19^F NMR signal with fluoromonomer content provides valuable information on how copolymer structure affects MR properties, regardless of fluorine content. This slope depends on the aforementioned chemical shift broadening due to sequence irregularity and on the fluorine detectability factor (Eq. (4), signal attenuated by aggregation, for example). In this study, the copolymer *T*_2_ relaxation times were significantly higher than TE times, so they did not play a major role.

HEAM-containing P_1_ copolymers have the highest slopes, reflecting superior ^19^F MR performance. Inversely, copolymers with the bulky comonomer THAM (P4) and with the strongly polar comonomers CBAM (P5) and MSEAM (P6) displayed peak broadening, accounting for their inferior ^19^F NMR performance. Surprisingly, though, copolymers with the strongly polar zwitterionic (P5) and sulfoxide (P6) monomers showed worse ^19^F NMR signal intensities than other copolymers at similar fluorine contents. In addition to the structural irregularity effect, their poor ^19^F NMR performance may be attributed to strong coulombic and dipole-dipole interactions between these ionic monomers.

Strong inter- and/or intramolecular interactions restrict chain mobility, reducing the detectability of the fluorine nuclei in Eq. (4). For zwitterionic fluorinated copolymers, single-chain nanoparticle (SCNP) formation is known to restrict mobility, further weakening the signal. Suggesting the formation of SCNPs due to strong intramolecular interactions, DLS measurements (Fig. S14) revealed that P5 copolymers have smaller hydrodynamic sizes than their counterparts in the other series, except for P5_80_. For this copolymer, intermolecular interactions prevailed over intramolecular interactions, leading to loose aggregates and decreasing the magnetic detectability (ν) of fluorines. Therefore, the signal intensities were even lower than expected for all zwitterionic P5 copolymers.

**Fig. 3:**
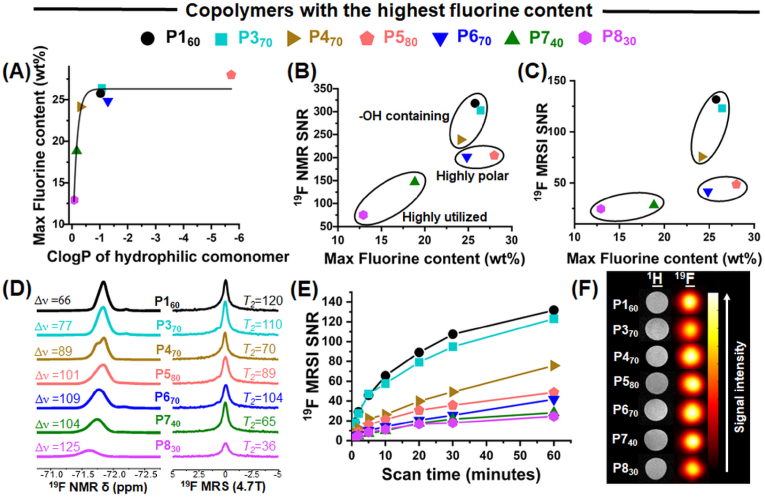
Characterization of ^19^F properties of water-soluble copolyacrylamides with the highest fluorine content of each series, namely P1_60_, P3_70_, P4_70_, P5_80_, P6_70_, P7_40_ and P8_30_, showed that P1_60_ exhibited the best performance. (A) Effect of comonomer hydrophilicity and (B) variation of ^19^F NMR (400 MHz) SNR and (C) ^19^F MRSI (4.7 T) SNR as a function of maximum fluorine content. (D) ^19^F NMR spectra with half-width values and ^19^F MRS (4.7 T) spectra with *T*_*2*_ values of copolymers at *c*_pol_ = 10 mg mL^−1^ in water. (E) ^19^F MRSI sensitivity assessment of copolymers based on SNR quantification as a function of acquisition duration. (F) ^1^H MRI and ^19^F MRSI of the copolymers at *c*_pol_ = 10 mg mL^−1^ with a 30-min scan time.

The maximum fluorine content achievable in water-soluble copolymers increased with the hydrophilicity of the comonomer, as indicated by cLogP values, reaching a plateau at 27 wt% fluorine (Fig. 3A). This trend aligns with reports on other water-soluble fluorinated polymers [20,23]. Based on these results, we selected the water-soluble copolymers of each series with the highest fluorine content for a more detailed analysis to identify optimal candidates for ^19^F MRI applications.

The simplest hydroxyl-containing copolymer, P1_60_ (P(TFEAM_60_-*stat*-HEAM_40_)), showed the best ^19^F NMR signal intensity and relaxation times, followed by dihydroxyl-containing P3_70_, showing the positive effect of hydroxyl groups on ^19^F MR SNR (Fig. 3B). Conversely, despite having three hydroxyl groups, P4_70_ underperformed, potentially due to steric hindrance of its bulky structure and to its lower hydrophilicity. Both the zwitterionic P5_80_ (with the highest fluorine content) and sulfoxide-containing P6_70_ (with fluorine nearly as high as that of P1_60_ and P3_70_) also underperformed, providing significantly lower signal intensity because ionic copolymers decrease the magnetic detectability (ν) of fluorines, as discussed above. Despite similar MR properties, the main benefit of P1_60_ over P3_70_ consists in a more straightforward copolymer synthesis using commercially available HEAM comonomer.

As expected, P7_40_ and P8_30_ yielded weaker ^19^F NMR SNR than the other copolymers due to their low fluorine content, resulting from their lower hydrophilicity. All ^19^F MRS (4.7 T) measurements matched all ^19^F NMR (400 MHz) results (Fig. 3B–S15). Furthermore, all ^19^F MRSI SNR values of all copolymers matched the previous measurements, except for P8_30_. It significantly outperformed but was, nevertheless, one of the two copolymers with the lowest ^19^F NMR signal intensities (Fig. 3C–S16).

When tested for their ^19^F relaxation properties (Fig. 3D), all copolymers with well-defined NMR peak shapes displayed high *T*_2_ values, which may compensate for clinical imaging sequences with longer echo times. These values ranged from 36 ms for P8_30_ to 120 ms for P1_60_, significantly exceeding the echo times (TE). Consequently, *T*_2_ relaxation times have negligible effect on the ^19^F NMR signal intensities (Eq. (4)). Instead, the intensities were primarily influenced by the fluorine content, degree of peak broadening, and the detectability factors, underscoring the importance of these parameters for MR performance of our systems. Regarding the *T*_*1*_ relaxation times, we observed the shortest value of 195 ms for P2_10_ and the longest of 485 ms for P6_70_ (Fig. S12). A shorter *T*_*1*_ theoretically enables the shortening of repetition time (TR) without a signal loss, allowing for more signal averaging within the same scan duration. Therefore, both *T*_*2*_ and *T*_*1*_ relaxation times must be considered as they both influence the overall sensitivity and efficiency of the imaging process. Lastly, all copolymers provided highly sensitive ^19^F MRS images at 10 mg mL^−1^ *c*_pol_ with a 30-min scan time (Fig. 3E and F). Considering all factors, the P1_60_ copolymer was selected for further experiments.

### 3.4 Comparison with previously reported hydrophilic fluorinated polymers

**Fig. 4:**
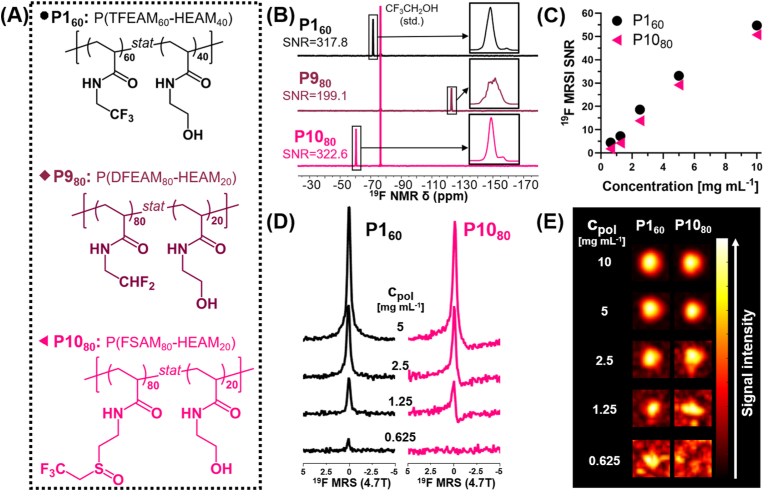
P1_60_ can be visualized by ^19^F MRI at a sub-mg mL^−1^ concentration, outperforming the ^19^F MR (4.7 T) sensitivity of the P10_80_ copolymer. (A) Structural representation of the three copolymers. (B^19^F NMR spectra of the copolymers with the highest fluorine contents. (C) Variation of ^19^F MRSI signal-to-noise ratio (SNR) as a function of copolymer concentration. (D) ^19^F MRS and (E) ^19^F MRSI results at different polymer concentrations, ranging from 0.625 mg mL^−1^ to 10 mg mL^−1^, with a 20-min acquisition time.

P1_60_ was compared with two copolymers based on recently reported thermoresponsive fluorinated acrylamides, namely *N*-(2,2-difluoroethyl)acrylamide (DFEAM) and *N*-(2-((2,2,2-trifluoroethyl)sulfinyl)ethyl)acrylamide (FSAM) (Fig. 4A). Both of these homopolymers are water-soluble below their lower critical solution temperatures (LCSTs), which are approximately 22 °C for PDFEAM [27] and 26 °C for PFSAM [20]. Both polymers display good ^19^F MRI properties due to polymer hydration even when aggregated, but to obtain soluble polymers in the biologically relevant temperature range (from room temperature to 37 °C), they were copolymerized with the previously optimized hydrophilic monomer HEAM. This approach increased their LCST, thereby yielding water-soluble copolymers (LCST > 37 °C) with a high fluorine content of up to 22–25 wt%.

For all three copolymers, ^19^F NMR SNR increased linearly with fluorine content. (Fig. S17A). We further compared the polymers with the highest fluorine content of each copolymer group, namely P1_60_, P9_80_, and P10_80_, based on their ^19^F NMR and MRI properties. P1_60_ and P10_80_ displayed similar results, with sharp singlet peaks, whereas the difluoro-substituted P9_80_ provided a lower ^19^F NMR signal intensity due to ^19^F NMR signal splitting by geminal hydrogen (Fig. 4B). Lastly, *in vitro* ^19^F MRI measurements in phantoms yielded images with high-quality signals consistent with NMR signal intensities (Fig. S17B).

The best-performing copolymers, P1_60_ (P(TFEAM_60_-HEAM_40_)) and P10_80_ (P(FSAM_80_-HEAM_20_)), were tested for their ^19^F MR sensitivity (Fig. 4C) to identify the lowest copolymer concentration still detectable by ^19^F MRI within 20 min, a reasonable acquisition time (Fig. 4D and E). Both copolymers were detected at significant dilutions, but P1_60_ outperformed P10_80_ in sensitivity as it was observable at *c*_pol_ = 0.625 mg mL^−1^ (corresponding to [F] = 8 mM), while the latter at *c*_pol_ = 1.25 mg mL^−1^ (corresponding to [F] 15 mM). The optimized P1_60_ is the first water-soluble fluoropolymer visualized by ^19^F MRI at a sub-mg mL^−1^ concentration (Fig. S18). Interestingly, both copolymers P1_60_ and P10_80_ showed a remarkable ability to prevent undesirable interactions with blood plasma (Table S11, Fig. S19, see Supporting *Information* for further details on antifouling properties of tested polymers), which could trigger an immune system response leading to faster clearance and compromised biocompatibility. Considering its excellent ^19^F MRI performance and easier monomer synthesis than that of P10_80_, HEAM-containing P1_60_ was selected for biological experiments.

### 3.5 In vivo tumor imaging with optimized fluoropolymer

Prior to *in vivo* administration, P1_60_ was tested for cytotoxicity by incubating 4T1 breast cancer cells with this polymer at concentrations ranging from 10 to 20 mg mL^−1^ for 24 h and performing the AlamarBlue cell viability assay (Fig. S20). In comparison with untreated controls (without polymer), no signs of cytotoxicity were observed, even at the highest copolymer concentrations (20 mg mL^−1^). Further, we tested the blood compatibility of P1_60_ by the hemolysis assay, and the polymer induced no hemolysis in the whole concentration range tested (up to 10 mg mL^−1^) after 6 h, respectively 12 h incubation at 37 °C (Fig. S21). Considering its excellent ^19^F MR properties and biocompatibility, the P1_60_ copolymer was deemed suitable as a^19^F MRI tracer for *in vivo* 4T1 solid tumor imaging in murine models.

For this experiment, a longer P1_60_ copolymer (DP 300) was synthesized (*M*_n_ = 51.7 kDa, *D*_h_ = 8.5 nm) for efficient passive accumulation in the tumor by enhanced permeation and retention (EPR) effect. In addition, the fluorescent label Cy7 was covalently attached for multimodal imaging by ^19^F MRI and fluorescence. Notably, the ^19^F relaxation times of the P1_60_-Cy7 tracer were nearly identical to those of the regular P1_60_ polymer, indicating that the incorporation of the Cy7 dye does not alter the polymer's relaxation properties. (Fig. S22). The resulting P1_60_-Cy7 tracer was intravenously injected into the bloodstream via the retro-orbital sinus (100 μL, *c*_pol_ = 70 mg mL^−1^, corresponding to [F] = 921 mM).

**Fig. 5:**
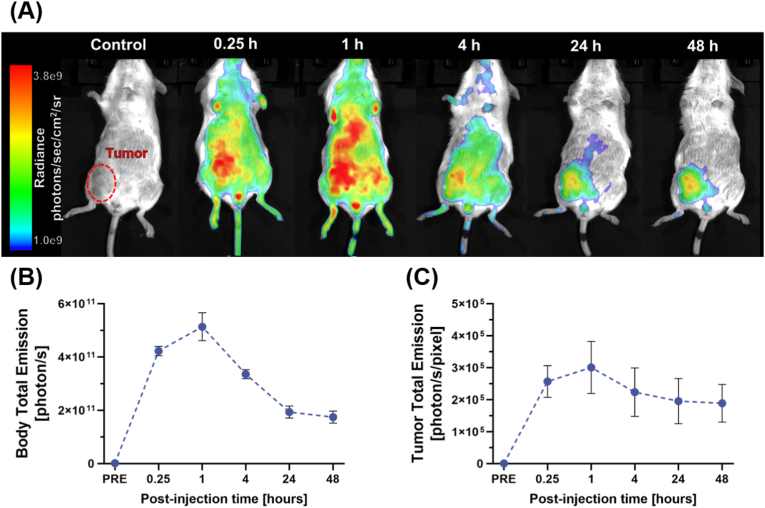
*In vivo* fluorescence imaging of P1_60_-Cy7 in 4T1 tumor-bearing mice before and after intravenous tracer injection shows polymer accumulation in the tumor. (A) The red dotted oval in the lower right abdomen outlines the tumor, revealing a high emission depot in the tumor over time due to tracer accumulation and systematic release from other body tissues. (B) Tracer total emission decreases over time due to clearance. (C) Tumor total emission stabilizes 24 h PI.

Initial fluorescence distribution measurements, taken 15 min and 1 h post-injection (PI), revealed that the tracer was spread throughout vascularized body tissues, albeit predominantly in the tumor region. At the 4-h PI time point, strong fluorescence signals were mainly restricted to the tumor site, and in subsequent imaging at 24- and 48-h PI, fluorescence signals were primarily localized in the tumor and its immediate area (Fig. 5A).

Semiquantitative fluorescence distribution analysis showed that whole-body emission increased in the first hours PI, eventually decreasing due to renal and biliary clearance. The marginal difference in emissions between the 24-h and 48-h PI time points may be attributed to tracer accumulation in the tumor (Fig. 5B). After tumor size normalization, this observation is supported by minimal changes in total emission from the tumor after 4 h PI (Fig. 5C). This is further confirmed by the gradual increase in the tumor-to-body ratio of total emission over time, which reached 19.3 ± 3.9 % (Fig. S23). This selective accumulation confirms that the P1_60_-Cy7 tracer can passively target tumor tissue. To validate this observation, we conducted control measurements on a healthy mouse, which initially exhibited a similar pattern of tracer distribution across vascularized tissues. However, unlike the tumor-bearing mice, the tracer gradually decreased throughout the body due to clearance mechanisms. (Figs. S24 and S25).

**Fig. 6:**
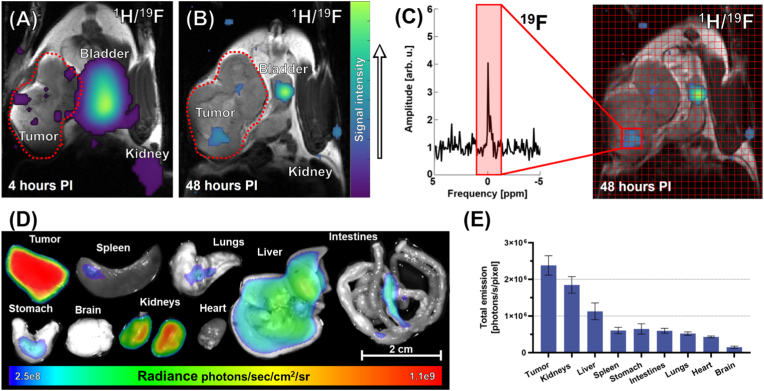
^19^F MR *in vivo* hotspot imaging (scan time = 30 min per ^19^F MRSI scan) and *ex vivo* biodistribution performed using fluorescence imaging show that P1_60_-Cy7 accumulates inside the tumor. (A) Overlaying reference ^1^H MRI with ^19^F MRSI 4 h PI reveals multiple depots inside the tumor (red dotted shape) and a hyperintense signal in the bladder (center) and kidney (bottom right). (B) ^1^H/^19^F image overlays 48 h PI demonstrates that the tracer is still located in the tumor despite renal clearance, indicated by the reduced signal in the bladder. (C) Summation of 9 individual spectra spatially corresponding to ^19^F MRSI voxels located inside the tumor tissue (right), further confirming polymer accumulation in the tumor. Distribution of the tracer P1_60_-Cy7 assessed by (D) *ex vivo* fluorescence and (E) normalized total emission from vital organs and tumor. All organs and tumors were extracted 48 h PI.

Although these initial *in vivo* fluorescence imaging findings provided valuable insights, fluoropolymer synthesis optimized for ^19^F MR hotspot imaging was the main goal of this study. Furthermore, optical fluorescence imaging, with its high sensitivity and ability to provide whole-body imaging in a single scan, may overestimate signals in subcutaneous tissues, such as tumors, due to its limited light penetration depth. In contrast, ^19^F MRI provides a more accurate representation of tracer distribution throughout the body. For those reasons, the fluorescence results were validated by MRI, an imaging method that holds high clinical significance despite its lower sensitivity compared to fluorescence imaging. To increase MR sensitivity, we restricted the FOV only to the area immediately surrounding the tumor tissue and conducted MR measurements at critical time points (4, 24, and 48 h PI) for comprehensive validation. Polymer signals in tumor tissues were detected by ^19^F MRSI (Fig. 6A, B, and C). Notably, in our *in vivo* experiments (Fig. S27), the tumor SNR after 48 h exceeded value 3 (commonly accepted minimum detection threshold for reliable ^19^F MRI signal identification) in two mice but measured around 2 in one mouse, illustrating minor inter-animal variability in signal detection. The sufficiently large difference in chemical shifts between our tracer and isoflurane used for anesthesia (approximately 8 ppm) allowed us to unambiguously distinguish and visualize the polymer, ensuring the reliability of MR-based detection (Fig. S26).

^19^F MR *in vivo* analysis effectively illustrates dynamic clearance. Both kidney and urinary bladder manifest a strong signal 4 h PI, indicating renal excretion of the copolymer. At the 48-h time point, the bladder signal was still noticeable, albeit weaker. These changes in bladder signal confirm P1_60_-Cy7 clearance.

The biodistribution of P1_60_-Cy7 was comprehensively analyzed by extracting (48 h PI) the tumor and vital organs, including the liver, spleen, lungs, intestines, stomach, brain, kidneys, and heart, for *ex vivo* fluorescence imaging (Fig. 6D). To improve the accuracy of the quantitative analysis and, thus, reduce systematic errors, total emission values were normalized to tissue pixel perimeter (Fig. 6E). Based on the results from this *ex vivo* analysis, the strongest fluorescence signal consistently derived from tumor tissue, in tumor-bearing mice, further validating the EPR effect and highlighting the ability of the P1_60_-Cy7 tracer to passively target the tumor microenvironment.

The strong fluorescence signal in kidneys may be attributed to renal clearance, in line with our ^19^F MRSI data (Fig. 6D and E). The signal is weaker in the liver despite its large mass, suggesting that reticuloendothelial excretion is a minor excretion mechanism, as with other water-soluble polymers. The fluorescence signal from the lungs is explained by its highly permeable vasculature and by the first-pass effect [34]. In contrast, the relatively minimal accumulation of the tracer in the brain and heart is associated with the blood-brain barrier and relatively low uptake of foreign substances, respectively [35]. Moreover, *ex vivo* ^19^F MRS imaging of the tumor, liver, and spleen (Fig. S27) strongly correlates with fluorescence imaging (Figs. S28 and S29).

## 4 Conclusions

Screening water-soluble semifluorinated copolymers for ^19^F MR properties shows that acrylamides outperform acrylates by incorporating a higher fluorine content thanks to their higher hydrophilicity. Among hydrophilic acrylamide copolymers with distinct structural features, the comonomer structure strongly affects ^19^F signal performance, with hydroxyl-containing copolymers generating the strongest ^19^F signals. By contrast, copolymers with strong ionic interactions display lower magnetic detectability, resulting in weaker signal intensities despite high fluorine contents. In this series, P(TFEAM_x_-*stat*-HEAM_y_) rivals previously published thermoresponsive fluorinated polymers for its straightforward synthesis and excellent ^19^F performance. With detection limits at sub-mg mL^−1^ concentrations, P(TFEAM_x_-*stat*-HEAM_y_) emerges as the optimal candidate for further biological studies and *in vivo* tumor visualization based on promising results. This study elucidates the structure–signal intensity relationships of statistical semifluorinated copolymers, underscoring their potential as highly sensitive ^19^F MRI tracers. These findings could revolutionize diagnostic imaging and accelerate the development of novel drug delivery systems, theranostic platforms, and hydrogels.

## CRediT authorship contribution statement

**Tuba Ayça Tunca Arın:** Writing – original draft, Visualization, Validation, Methodology, Investigation, Formal analysis, Data curation. **Dominik Havlíček:** Writing – original draft, Visualization, Validation, Methodology, Investigation, Data curation. **Diego Fernando Dorado Daza:** Methodology, Investigation. **Natalia Jirát-Ziółkowska:** Methodology, Investigation. **Ognen Pop-Georgievski:** Writing – review & editing, Supervision, Project administration, Investigation, Funding acquisition. **Daniel Jirák:** Writing – review & editing, Supervision, Resources, Project administration, Methodology, Funding acquisition, Conceptualization. **Ondrej Sedlacek:** Writing – review & editing, Validation, Supervision, Project administration, Methodology, Conceptualization.

## Declaration of competing interest

The authors declare the following financial interests/personal relationships which may be considered as potential competing interests:Ognen Pop-Georgievski reports financial support was provided by 10.13039/501100001824Czech Science Foundation. Daniel Jirak reports financial support was provided by National Institute for Research of Metabolic and Cardiovascular Diseases. Daniel Jirak reports financial support was provided by 10.13039/501100003243Ministry of Health of the Czech Republic. Tuba Ayca Tunca Arin reports financial support was provided by Grant Agency of 10.13039/100007397Charles University. Dominik Havlicek reports financial support was provided by Grant Agency of 10.13039/100007397Charles University. If there are other authors, they declare that they have no known competing financial interests or personal relationships that could have appeared to influence the work reported in this paper.

## Data Availability

All data are available from corresponding authors upon reasonable request.
