# Supplementary material for: Water-soluble fluorinated copolymers as highly sensitive 19F MRI tracers: From structure optimization to multimodal tumor imaging
Source: Mater Today Bio. 2025 Jan 4;31:101462. doi: 10.1016/j.mtbio.2025.101462 (PMC11786703; doi:10.1016/j.mtbio.2025.101462)
Supplement: Multimedia component 1 [file mmc1.docx]

**Supporting information**

**Water-soluble fluorinated copolymers as highly sensitive ^19^F MRI tracers: From structure optimization to multimodal tumor imaging**

Tuba Ayça Tunca Arın,^a,+^ Dominik Havlíček,^b,c,+^ Diego Fernando Dorado Daza,^d^ Natalia Jirát-Ziółkowska,^b,c^ Ognen Pop-Georgievski,^d^ Daniel Jirák^b,e*^ and Ondrej Sedlacek^a*^

^a^Department of Physical and Macromolecular Chemistry, Faculty of Science, Charles University, 128 00 Prague 2, Czech Republic

^b^Department of Diagnostic and Interventional Radiology, Institute for Clinical and Experimental Medicine, 140 21 Prague 4, Czech Republic

^c^Institute of Biophysics and Informatics, First Faculty of Medicine, Charles University, Prague, 128 00 Prague 2, Czech Republic

^d^Department of Chemistry and Physics of Surfaces and Interfaces, Institute of Macromolecular Chemistry, AS CR, 162 06 Prague 6, Czech Republic

^e^Faculty of Health Studies, Technical University of Liberec, 461 17 Liberec, Czech Republic

**^+^**Equally contributing authors

E-mail: [sedlacek@natur.cuni.cz](mailto:sedlacek@natur.cuni.cz), [daniel.jirak@ikem.cz](mailto:daniel.jirak@ikem.cz)

**Preparation of polymer coatings**

All gold-coated substrates were obtained from the Institute of Photonics and Electronics, Czech Academy of Sciences. Optically thick gold layers (ca. 200 nm) were deposited on one side of polished silicon wafers (CZ, orientation <100>, *p*-doped, resistivity 5-20 Ω·cm, Siegert Wafer GmbH, Germany) with a 5-nm titanium adhesion layer. The wafers were cut into 2 × 1 cm^2^ pieces prior to further processing. The gold-coated substrates that were prepared for surface plasmon resonance (SPR) analysis consisted of a glass support, ~2 nm of a titanium adhesion layer, and ~50 nm of a gold layer. All substrates were cleaned by sonication in ethanol and water, blow-dried with nitrogen and activated in a UV-ozone cleaner for 20 min before immersion in polymer solutions. Polymer grafting proceeded under good solvent conditions, i.e., from 2 mg mL^-1^ ethanol solutions at room temperature for 60 h. Subsequently, all modified substrates were repeatedly washed with ethanol and distilled water and blow-dried in a stream of purified nitrogen.

**Characterization of polymer coatings**

*Spectroscopic ellipsometry (SE)* measurements in air were performed on a J.A. Woollam M-2000X spectroscopic ellipsometer operating in rotating compensator mode at a 60–70° angle of incidence (AOI) range (with a 5° step) and *λ* = 250−1000 nm spectral range. The data were fitted with CompleteEASE software using a multilayer model. The thickness and refractive index of dry and swollen polymer layers were calculated by simultaneous fitting of the obtained ellipsometric data using Cauchy dispersion functions. All thickness values are averages from 6 independent measurements, expressed as mean ± standard deviation.

*Calculation of surface-related parameters of polymer chains grafted to gold substrates.* The grafting density $\sigma=\frac{h_{\mathrm{dry}}\rho N_{A}}{M_{n}}$ and distance between grafting sites, assuming circular packing, $D=\sqrt{\frac{2}{\sqrt{3}\sigma}}$ were estimated based on the layer thickness in the dry state (*h*_dry_), as determined by ellipsometry, on the bulk densities of the polymers (1.1 g·cm^-3^), and on *N*_A_, which is the Avogadro constant. Assuming that the chains were random coils, the radius of gyration *R*_g_ of the polymers in water was calculated from the hydrodynamic radius [1]. The overlap parameter $\frac{D}{2R_{g}}$ was used to describe the state of tethered polymer chains, with (i) $\frac{D}{2R_{g}}>1.0$ indicating that the chains are in a “mushroom” state; (ii)$\frac{D}{2R_{g}}=1.0$, in a mushroom-to-brush transition state; and $\frac{D}{2R_{g}}<1.0$, in a brush conformation, stretch away from the surface.

*Contact angle goniometry.* The static water contact angles were measured with a contact angle goniometer OCA 20 (Dataphysics, Germany) equipped with SCA 21 software. For this purpose, 3 μL drops were deposited on test surfaces, and their contact angle values were determined using the tangent leaning method. All contact angle values are averages from 6 independent measurements, expressed as mean ± standard deviation.

*Surface plasmon resonance (SPR)****.*** Non-specific protein adsorption on PEG, P1_30_, P3_70_, P4_70_, P5_80_, P9_80,_ and P10_80_ coated and bare SPR chips was measured using an SPR instrument based on the Kretschmann geometry of the attenuated total reflection and spectral interrogation. In this system, the shift in the resonance wavelength was recorded, and the amount of the biomolecules adsorbed on the coating was estimated from the difference between the baselines in pure PBS (pH 7.4) before and after contact with undiluted human blood plasma (HBP). The solutions were pumped through a 4-channel flow cell attached to an SPR chip for 15 min. The measurements were performed at a flow rate of 25 μL·min^-1^ and a temperature of 25 °C (± 0.1 °C). In order to translate the surface refractive-index change measured via SPR to the surface mass density, an experimental factor of 18 ng·cm^-2^·nm^-1^ was used (valid at the wavelength of 750 nm).

**P1_x_ - P(TFEAM_x_-HEAM_y_)**

**Table S1**. Characteristics of P1_x_ copolymers


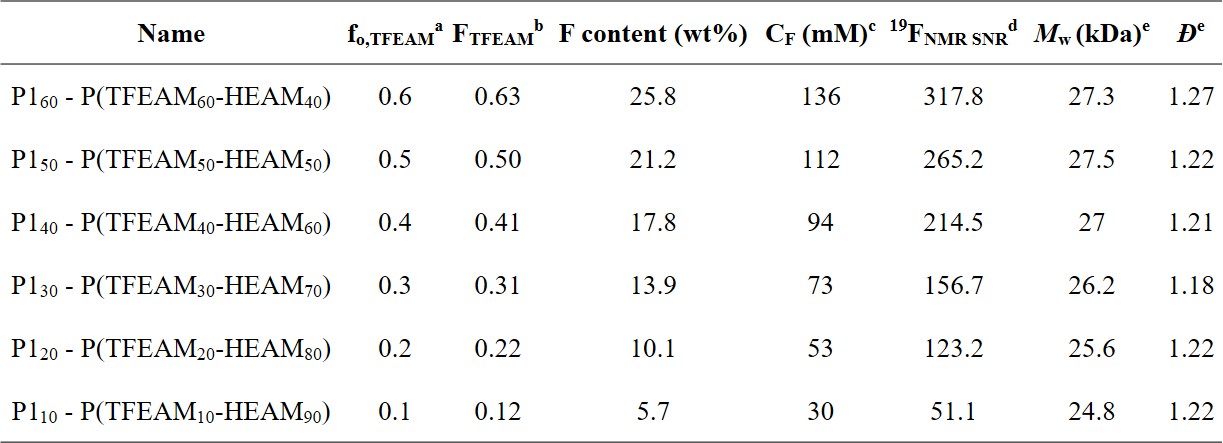


^a^ Theoretical TFEAM fraction in copolymers. ^b^ TFEAM fraction in copolymers determined by ^1^H NMR in DMSO-*d*_6_. ^c^ Molar concentration of fluorine in 10 mg mL^-1^ polymer solution. ^d^ Signal to noise ratios calculated using a built-in MestReNova function. ^e^ Determined by SEC in DMAc/LiCl.


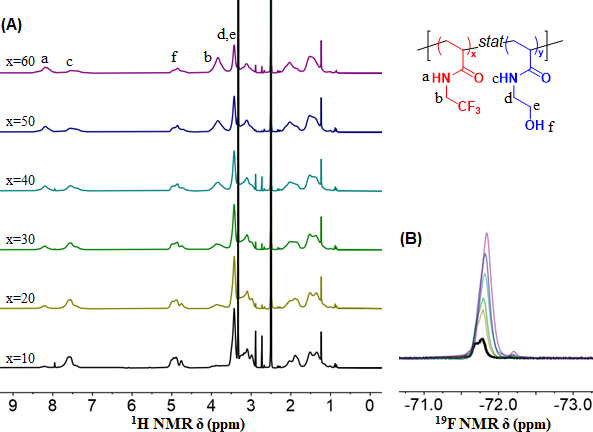


**Figure S1.** (A) ^1^H NMR spectra of P(TFEAM_x_-HEAM_y_) in DMSO-*d*_6_. (B) ^19^F NMR spectra of P(TFEAM_x_-HEAM_y_) in H_2_O/D_2_O (95/5 v/v).

**P2_x_ - P(TFEA_x_-HEA_y_)**

**Table S2**. Characteristics of P2_x_ copolymers


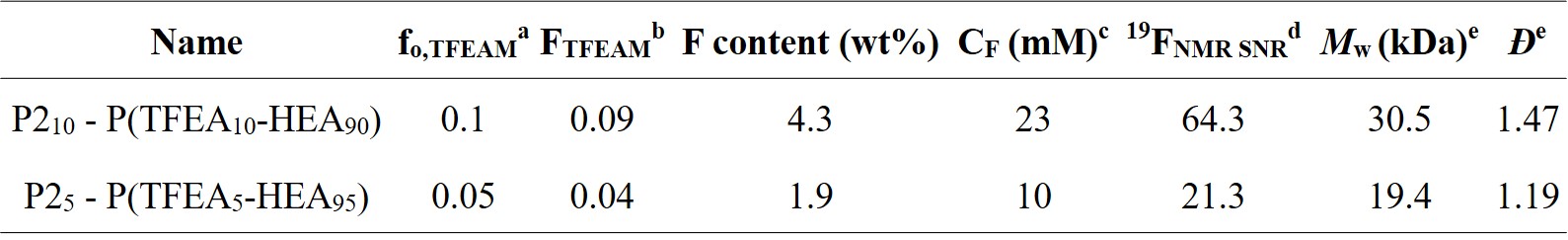


^a^ Theoretical TFEA fraction in copolymers. ^b^ TFEA fraction in copolymers determined by ^1^H NMR in DMSO-*d*_6_. ^c^ Molar concentration of fluorine in 10 mg mL^-1^ polymer solution. ^d^ Signal-to-noise ratios calculated using a built-in MestReNova function. ^e^ Determined by SEC in DMAc/LiCl.


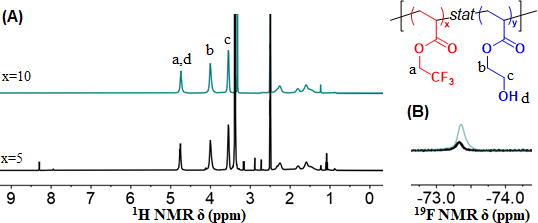


**Figure S2.** (A) ^1^H NMR spectra of P(TFEA_x_-HEA_y_) in DMSO-*d*_6_. (B) ^19^F NMR spectra of P(TFEA_x_-HEA_y_) in H_2_O/D_2_O (95/5 v/v).

**P3_x_ - P(TFEAM_x_-DHPAM_y_)**

**Table S3**. Characteristics of P3_x_ copolymers


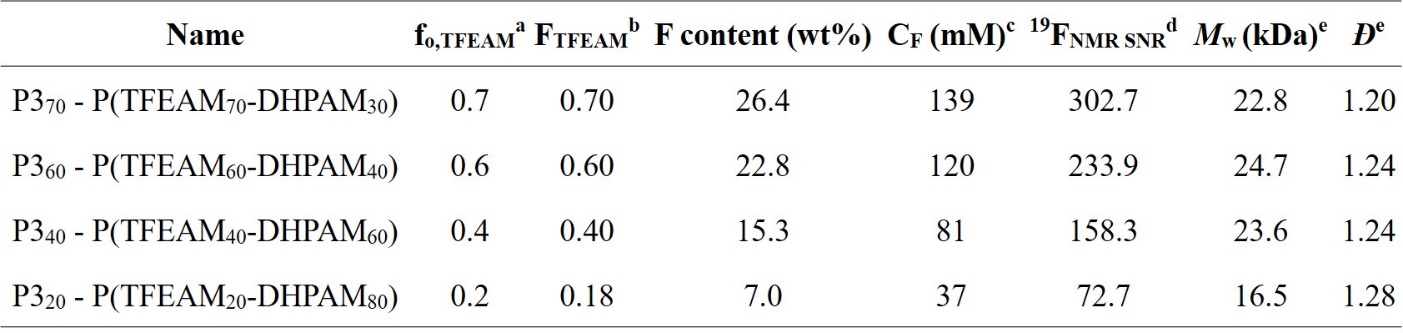


^a^ Theoretical TFEAM fraction in copolymers. ^b^ TFEAM fraction in copolymers determined by ^1^H NMR in DMSO-*d*_6_. ^c^ Molar concentration of fluorine in 10 mg mL^-1^ polymer solution. ^d^ Signal-to-noise ratios calculated using a built-in MestReNova function. ^e^ Determined by SEC in DMAc/LiCl.


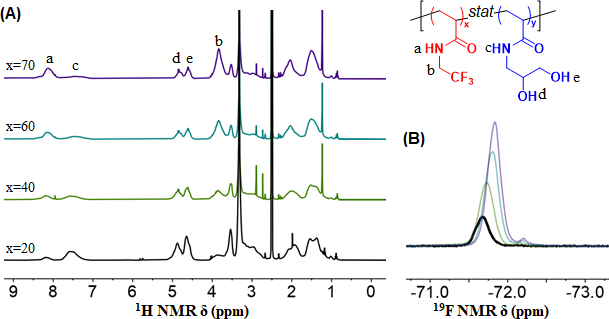


**Figure S3.** (A) ^1^H NMR spectra of P(TFEAM_x_-DHPAM_y_) in DMSO-*d*_6_. (B) ^19^F NMR spectra of P(TFEAM_x_-DHPAM_y_) in H_2_O/D_2_O (95/5 v/v).

**P4_x_ - P(TFEAM_x_-THAM_y_)**

**Table S4**. Characteristics of P4_x_ copolymers


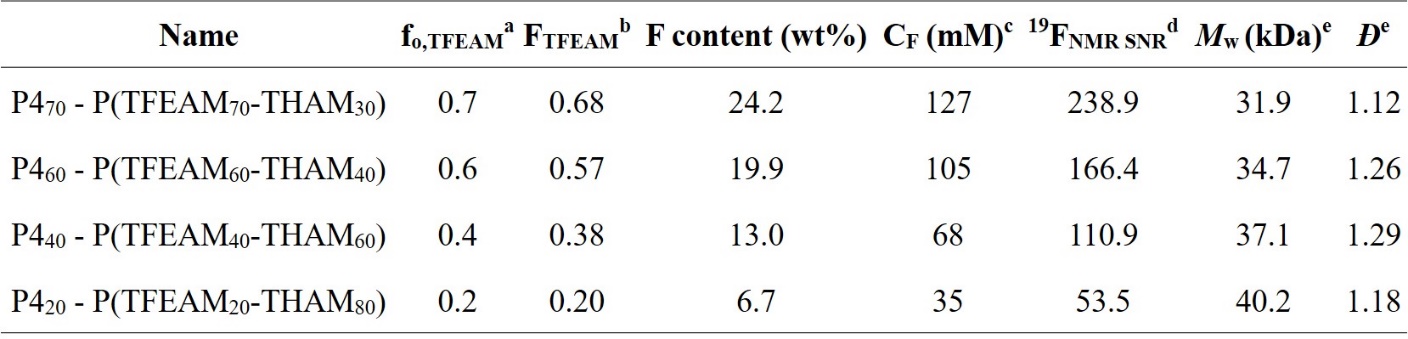


^a^ Theoretical TFEAM fraction in copolymers. ^b^ TFEAM fraction in copolymers determined by ^1^H NMR in DMSO-*d*_6_. ^c^ Molar concentration of fluorine in 10 mg mL^-1^ polymer solution. ^d^ Signal-to-noise ratios calculated using a built-in MestReNova function. ^e^ Determined by SEC in DMAc/LiCl.


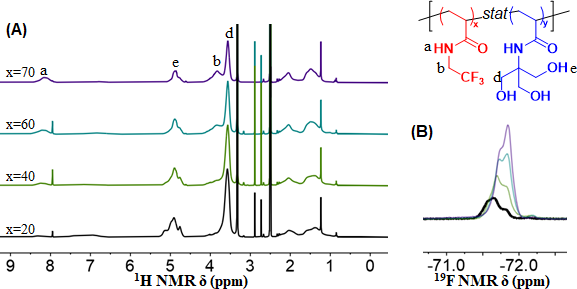


**Figure S4.** (A) ^1^H NMR spectra of P(TFEAM_x_-THAM_y_) in DMSO-*d*_6_. (B) ^19^F NMR spectra of P(TFEAM_x_-THAM_y_) in H_2_O/D_2_O (95/5 v/v).

**P5_x_ - P(TFEAM_x_-CBAM_y_)**

**Table S5**. Characteristics of P5_x_ copolymers


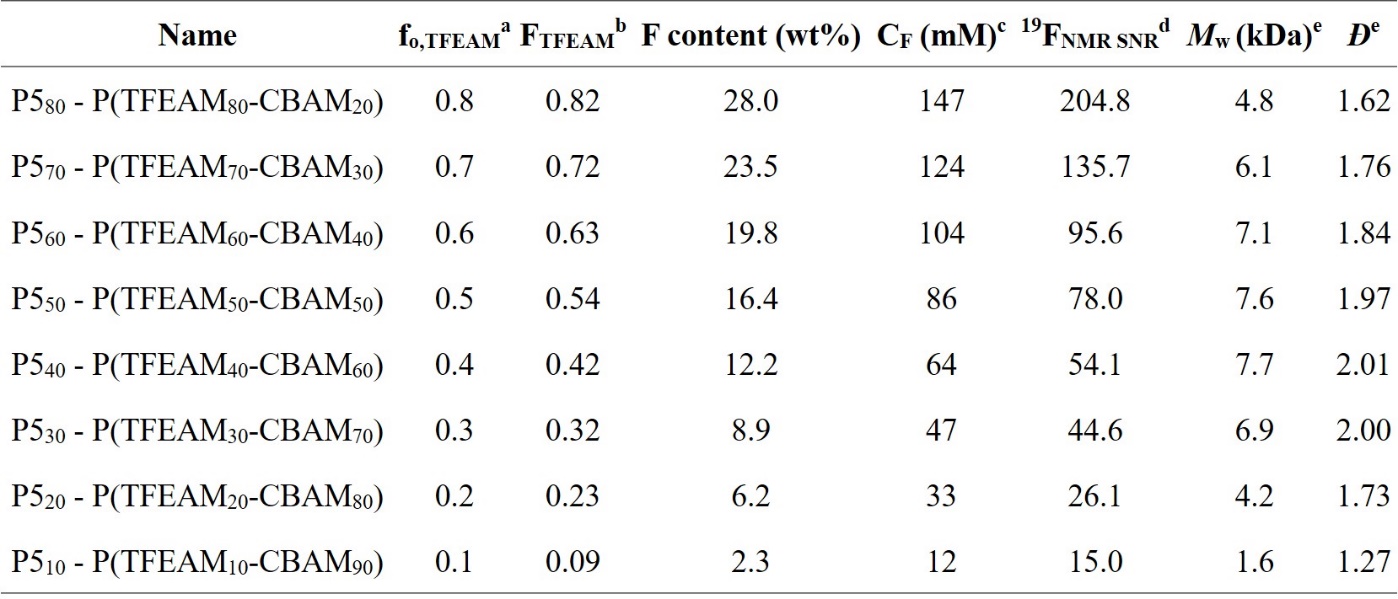


^a^ Theoretical TFEAM fraction in copolymers. ^b^ TFEAM fraction in copolymers determined by ^1^H NMR in D_2_O. ^c^ Molar concentration of fluorine in 10 mg mL^-1^ polymer solution. ^d^ Signal-to-noise ratios calculated using a built-in MestReNova function. ^e^ Determined by SEC in a methanol/buffer mixture (80:20 v/v).


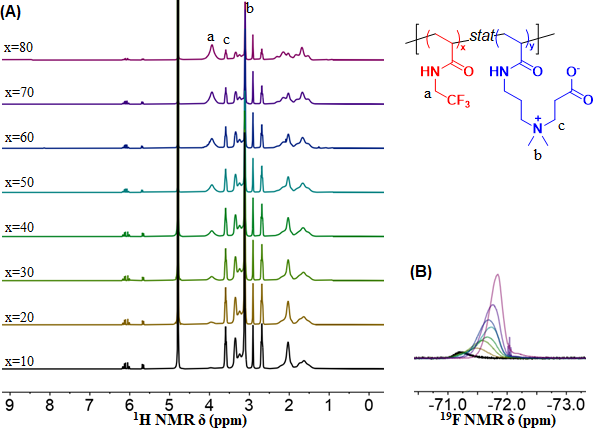


**Figure S5.** (A) ^1^H NMR spectra of P(TFEAM_x_-CBAM_y_) in D_2_O. (B) ^19^F NMR spectra of P(TFEAM_x_-CBAM_y_) in H_2_O/D_2_O (95/5 v/v).

**P6_x_ - P(TFEAM_x_-MSEAM_y_)**

**Table S6**. Characteristics of P6_x_ copolymers


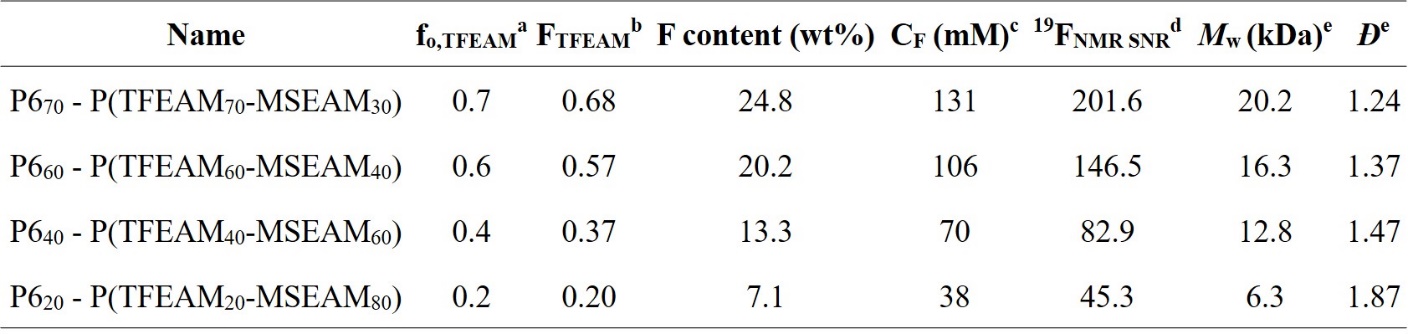


^a^ Theoretical TFEAM fraction in copolymers. ^b^ TFEAM fraction in copolymers determined by ^1^H NMR in DMSO-*d*_6_. ^c^ Molar concentration of fluorine in 10 mg mL^-1^ polymer solution. ^d^ Signal-to-noise ratios calculated using a built-in MestReNova function. ^e^ Determined by SEC in DMAc/LiCl.


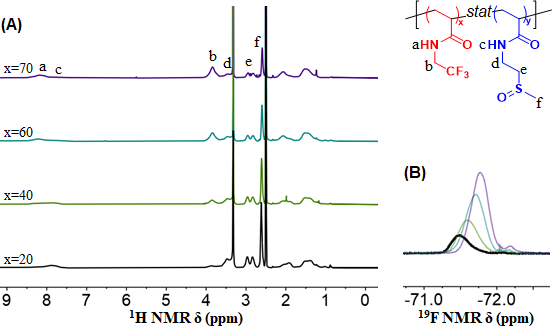


**Figure S6.** (A) ^1^H NMR spectra of P(TFEAM_x_-MSEAM_y_) in DMSO-*d*_6_. (B) ^19^F NMR spectra of P(TFEAM_x_-MSEAM_y_) in H_2_O/D_2_O (95/5 v/v).

**P7_x_ - P(TFEAM_x_-DMAM_y_)**

**Table S7**. Characteristics of P7_x_ copolymers


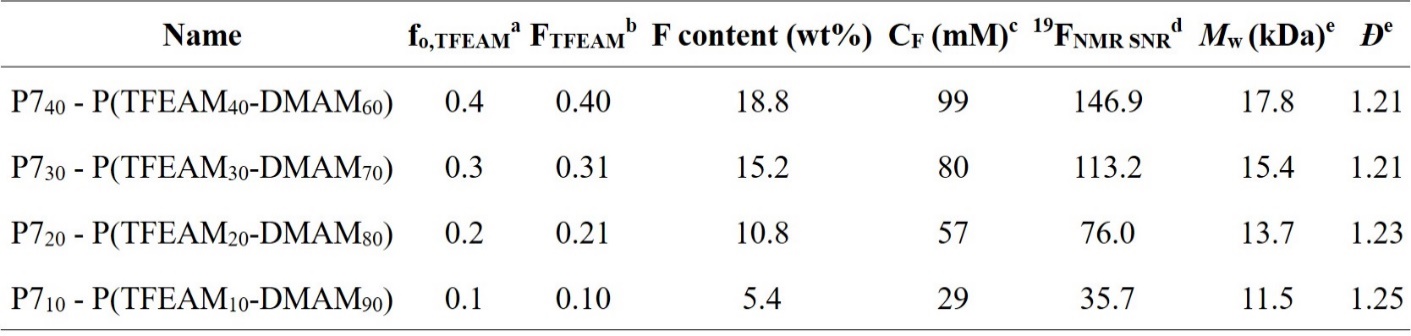


^a^ Theoretical TFEAM fraction in copolymers. ^b^ TFEAM fraction in copolymers determined by ^1^H NMR in DMSO-*d*_6_. ^c^ Molar concentration of fluorine in 10 mg mL^-1^ polymer solution. ^d^ Signal-to-noise ratios calculated using a built-in MestReNova function. ^e^ Determined by SEC in DMAc/LiCl.


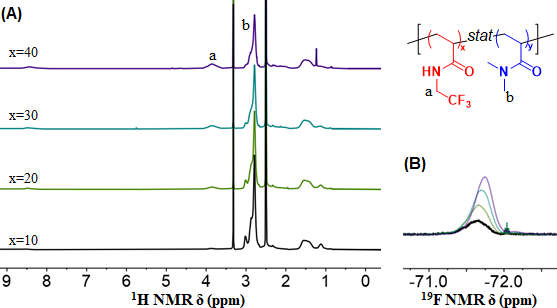


**Figure S7.** (A) ^1^H NMR spectra of P(TFEAM_x_-DMAM_y_) in DMSO-*d*_6_. (B) ^19^F NMR spectra of P(TFEAM_x_-DMAM_y_) in H_2_O/D_2_O (95/5 v/v).

**P8_x_ - P(TFEAM_x_-NAM_y_)**

**Table S8**. Characteristics of P8_x_ copolymers


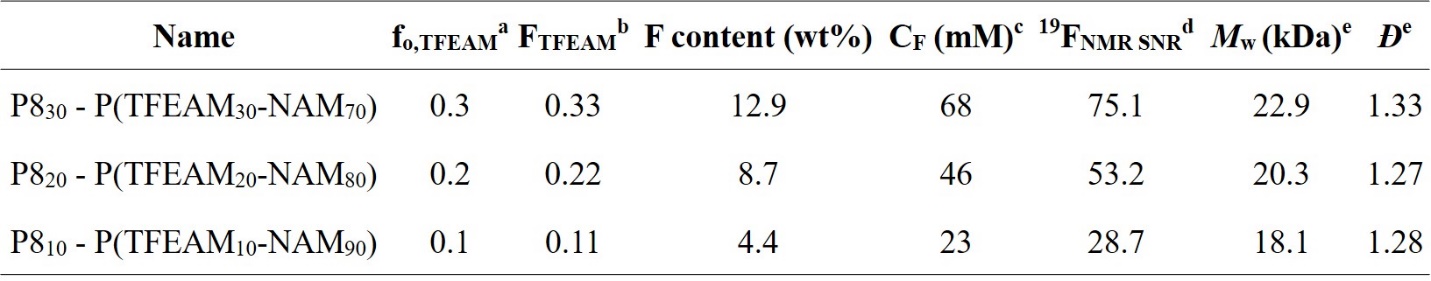


^a^ Theoretical TFEAM fraction in copolymers. ^b^ TFEAM fraction in copolymers determined by ^1^H NMR in DMSO-*d*_6_. ^c^ Molar concentration of fluorine in 10 mg mL^-1^ polymer solution. ^d^ Signal-to-noise ratios calculated using a built-in MestReNova function. ^e^ Determined by SEC in DMAc/LiCl.


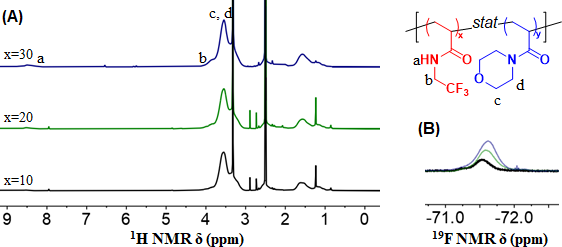


**Figure S8.** (A) ^1^H NMR spectra of P(TFEAM_x_-NAM_y_) in DMSO-*d*_6_. (B) ^19^F NMR spectra of P(TFEAM_x_-NAM_y_) in H_2_O/D_2_O (95/5 v/v).

**P9_x_ - P(DFEAM_x_-HEAM_y_)**

**Table S9**. Characteristics of P9_x_ copolymers


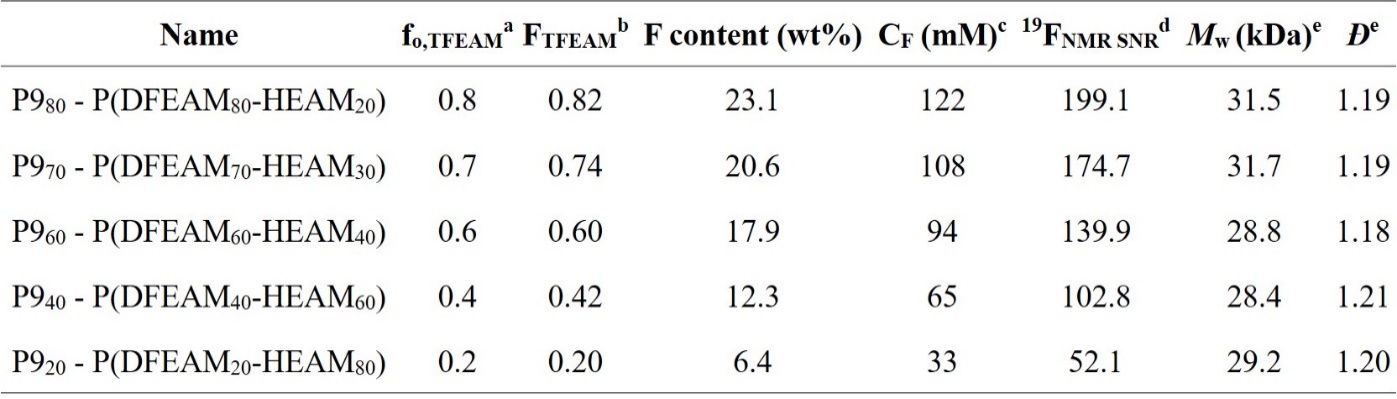


^a^ Theoretical DFEAM fraction in copolymers. ^b^ DFEAM fraction in copolymers determined by ^1^H NMR in DMSO-*d*_6_. ^c^ Molar concentration of fluorine in 10 mg mL^-1^ polymer solution. ^d^ Signal-to-noise ratios calculated using a built-in MestReNova function. ^e^ Determined by SEC in DMAc/LiCl.


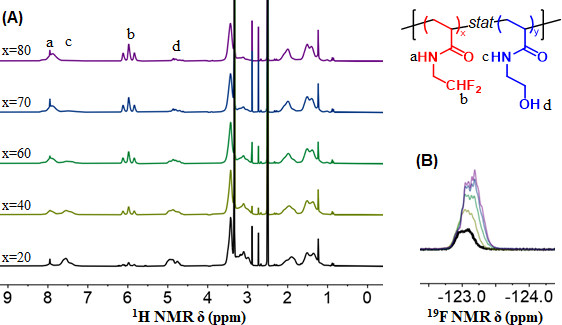


**Figure S9.** (A) ^1^H NMR spectra of P(DFEAM_x_-HEAM_y_) in DMSO-*d*_6_. (B) ^19^F NMR spectra of P(DFEAM_x_-HEAM_y_) in H_2_O/D_2_O (95/5 v/v).

**P10_x_ - P(FSAM_x_-HEAM_y_)**

**Table S10**. Characteristics of P10_x_ copolymers


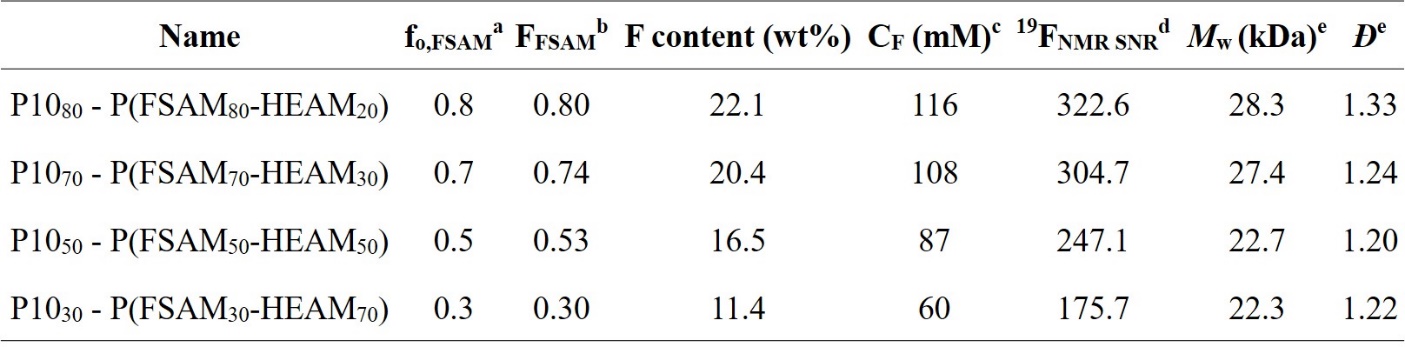


^a^ Theoretical FSAM fraction in copolymers. ^b^ FSAM fraction in copolymers determined by ^1^H NMR in DMSO-*d*_6_. ^c^ Molar concentration of fluorine in 10 mg mL^-1^ polymer solution. ^d^ Signal-to-noise ratios calculated using a built-in MestReNova function. ^e^ Determined by SEC in DMAc/LiCl.


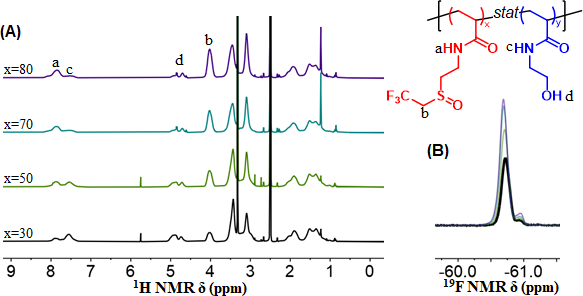


**Figure S10.** (A) ^1^H NMR spectra of P(FSAM_x_-HEAM_y_) in DMSO-*d*_6_. (B) ^19^F NMR spectra of P(FSAM_x_-HEAM_y_) in H_2_O/D_2_O (95/5 v/v).

**
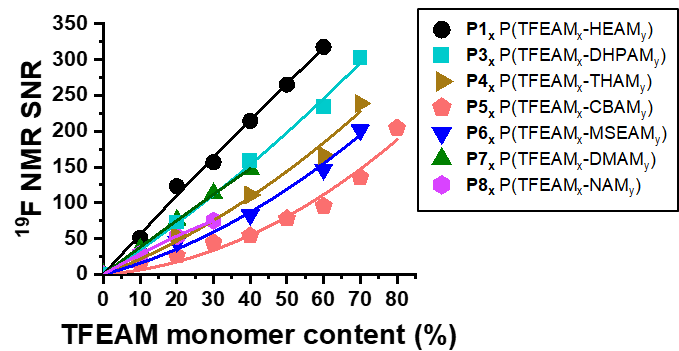
**

**Figure S11.** TFEAM molar content of all copolymer compositions.


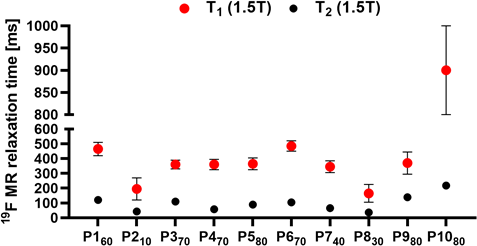


**Figure S12.** *T_1_* and *T_2_* relaxation times of semi-fluorinated polymers on a 4.7 T spectrometer at 20 °C.

**
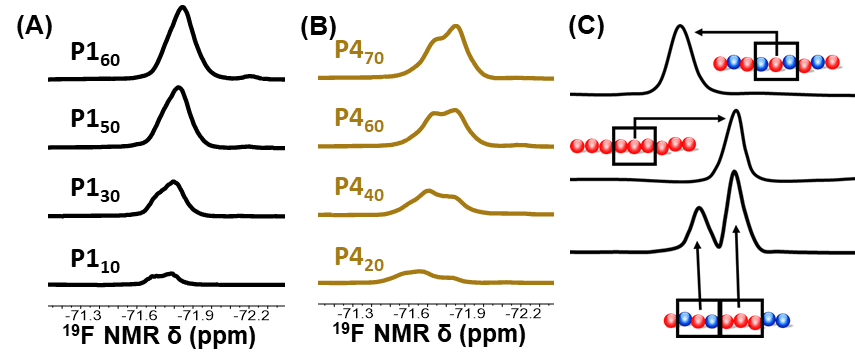
**

**Figure S13.** ^19^F NMR spectra of TFEAM copolymers with (A) HEAM and (B) THAM measured in water at *c*_pol_ = 10 mg mL^-1^. (C) Graphical explanation of double peak formation.


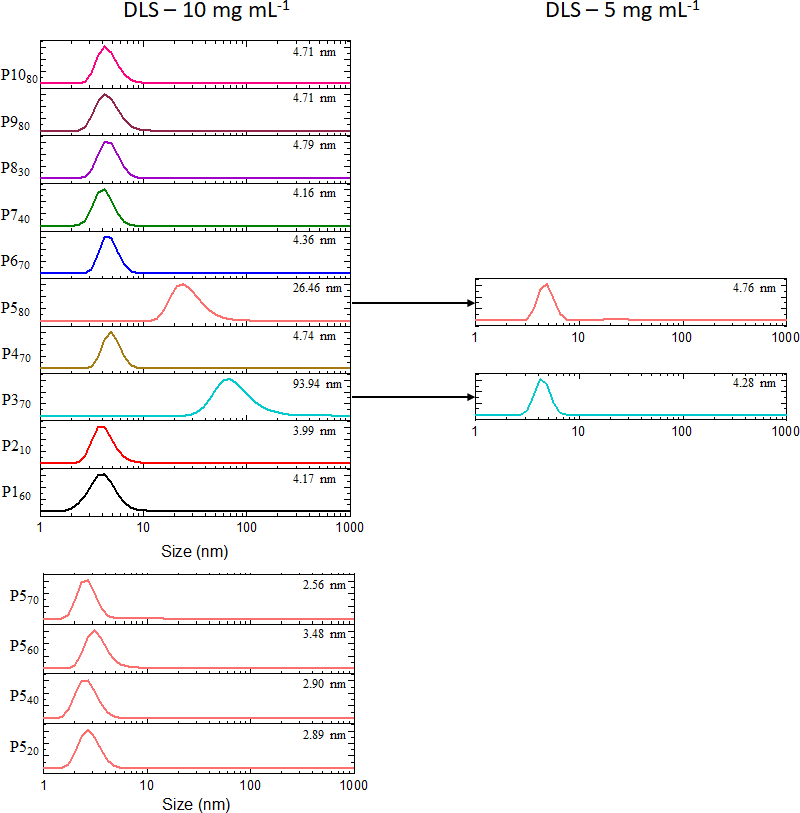


**Figure S14.** Volume-weighted DLS size distributions of copolymers in water (*c*_pol_ = 10 or 5 mg mL^–1^).


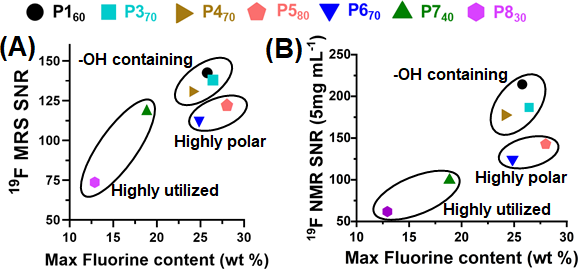


**Figure S15.** Variation of (A) ^19^F MRS SNR (4.7 T) (at *c*_pol_ = 10 mg mL^-1^) and (B) ^19^F NMR SNR (400 MHz) (at *c*_pol_ = 5 mg mL^-1^) as a function of maximum fluorine content.


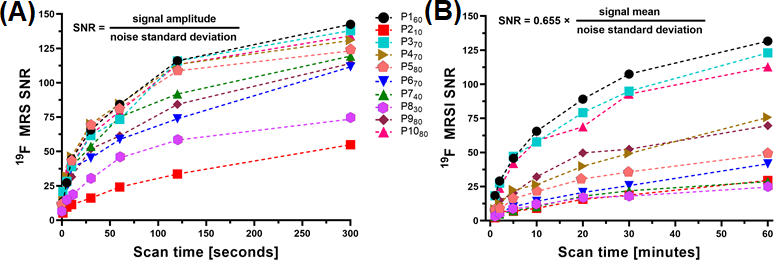


**Figure S16.** Phantom (A) ^19^F MRS and (B) ^19^F MRSI sensitivity assessment of the polymers based on SNR quantification as a function of measurement duration.


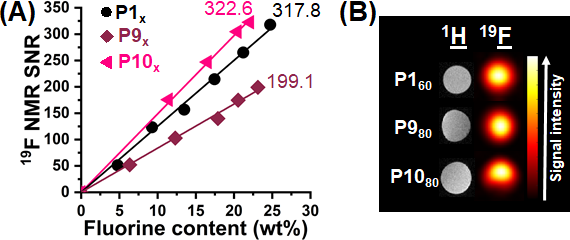


**Figure S17.** (A) Variation of ^19^F NMR SNR as a function of fluorine content (B) *In vitro* ^1^H and ^19^F MRSI of the copolymers at *c*_pol_ = 10 mg mL^-1^ and 30-minute acquisition time.


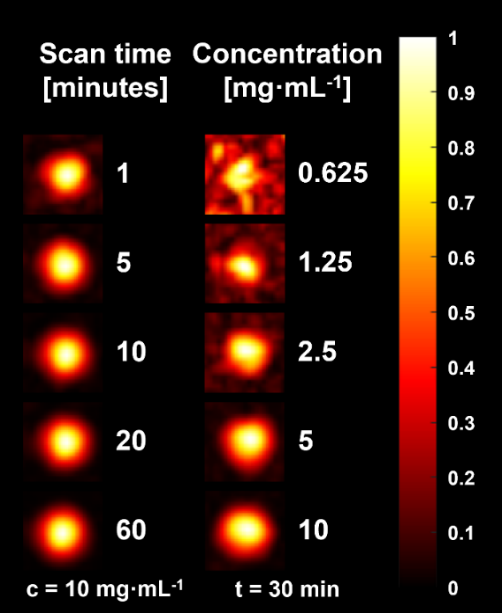


**Figure S18.** ^19^F MR sensitivity (4.7T) of the P1_60_ phantom sample (V = 500 µL): ^19^F MRSI of 10 mg mL^-^ ^1^ polymer at various scan times (left column) and 0.625 – 10 mg mL^-1^ polymer at 30 minutes scan time (right column).

**Antifouling properties of semifluorinated polyacrylamides**

The antifouling properties of polymers are crucial for developing new polymer biomaterials as they prevent undesirable interactions with blood plasma proteins, which could trigger an immune system response leading to faster clearance and compromised biocompatibility.

For this purpose, we grafted the selected water-soluble copolymers (P1_60_, P3_70_, P4_70_, P5_80_, P9_80,_ and P10_80_) to gold surfaces (via chain-end trithiocarbonate groups). PEG was utilized as a standard polymer layer, traditionaly used to demonstrate antifouling properties. The polymer brush thickness of the different surfaces was determined by spectroscopic ellipsometry and used to calculate the surface chain density (*σ*), the distance between grafting points (*D*), and the structural parameter *D*/2R_g_ of the surface anchored polymer chains in contact with water. Grafting proceeded under good solvent conditions, so the dry layer thickness ranged from 1.0 to 2.0 nm, with moderate surface chain densities [2,3] for copolymers with *σ* ranging between 0.03 – 0.06 chains/nm^2^ (**Table S11**). However, in all copolymers, the chains were attached to the gold surface in a brush configuration, given the low values of the structural overlap parameter *D/R_g_* (approximately 0.4). As shown by contact-angle goniometry, these brushes were only moderately hydrophilic due to the presence of fluorine atoms in the hydrophilic copolymers. Nevertheless, the zwitterionic copolymer P5_80_ and the reference PEG were much more hydrophilic than all other copolymers.

The antifouling properties of the grafted polymer chains exhibiting similar polymer brush conformation were determined by surface plasmon resonance (SPR) spectroscopy (**Figure S19**). The dihydroxy-containing P3_70_ copolymer revealed the best antifouling properties, reducing plasma protein binding to 4% of the control (bare gold surface) and outperforming analogous PEG brushes by an order of magnitude (43% fouling). Nevertheless, all other water-soluble fluoropolymers also outperformed PEG (9-18% fouling), except for the zwitterionic copolymer P5_80_. Eventhough P5_80_ was the most hydrophilic of all fluoropolymer coatings, it failed to prevent non-specific plasma protein adsorption. However, as previously noted, hydrophilicity is not the main prerequisite for fluoropolymer brushes to attain antifouling properties. The inferior antifouling properties of P5_80_ may be explained by intramolecular aggregation of its hydrophobic segments, predominantly with patchy-like structures in P5_80_ brush surfaces, thus insufficiently protecting it from plasma fouling. Accordingly, the P1_60_ copolymer showed both excellent antifouling and ^19^F MRI properties, which further justified its potential in the biomedical research.

**Table S11**. Surface properties of polymer brushes

|  | *h*_dry_ (nm) | *R*_h_(*ƞ*)^a^ (nm) | *R*_g_^b^ (nm) | *σ*^c^ (chain per nm^2^) | *D*^d^ (nm) | *D*/2*R*_g_^e^ | *θ*_static_^f^ (°) |
| --- | --- | --- | --- | --- | --- | --- | --- |
| PEO | 1.4±0.2 | 2.1 | 3.7 | 0.18 | 2.5 | 0.3 | 35± 2 |
| P1_60_ | 1.1±0.1 | 2.9 | 5.2 | 0.03 | 5.8 | 0.6 | 60 ± 1 |
| P3_70_ | 1.6±0.1 | 2.8 | 5.0 | 0.06 | 4.4 | 0.4 | 56 ± 1 |
| P4_70_ | 1.4±0.1 | 3.8 | 6.7 | 0.03 | 6.0 | 0.4 | 58 ± 2 |
| P5_80_ | 1.2±0.1 | 4.2 | 7.5 | 0.04 | 5.4 | 0.4 | 62 ± 2 |
| P9_90_ | 2.1±0.1 | 3.2 | 5.7 | 0.05 | 4.7 | 0.4 | 39 ± 1 |
| P10_80_ | 1.7±0.1 | 3.0 | 5.3 | 0.05 | 4.6 | 0.4 | 57 ± 1 |

^a^ Hydrodynamic radius measured by viscosimetry. ^b^ Radius of gyration assuming that the polymer chains are random coils, i.e., *R*_g_ = 1.78 × *R*_h_. ^c^ Surface chain density. ^d^ Distance between grafting sites assuming that polymer chains show hexagonal packing. ^e^ Structural overlap parameter indicating that the polymer chains display a brush configuration when smaller than 1.0. ^f^ Static water contact angle measured by goniometry.


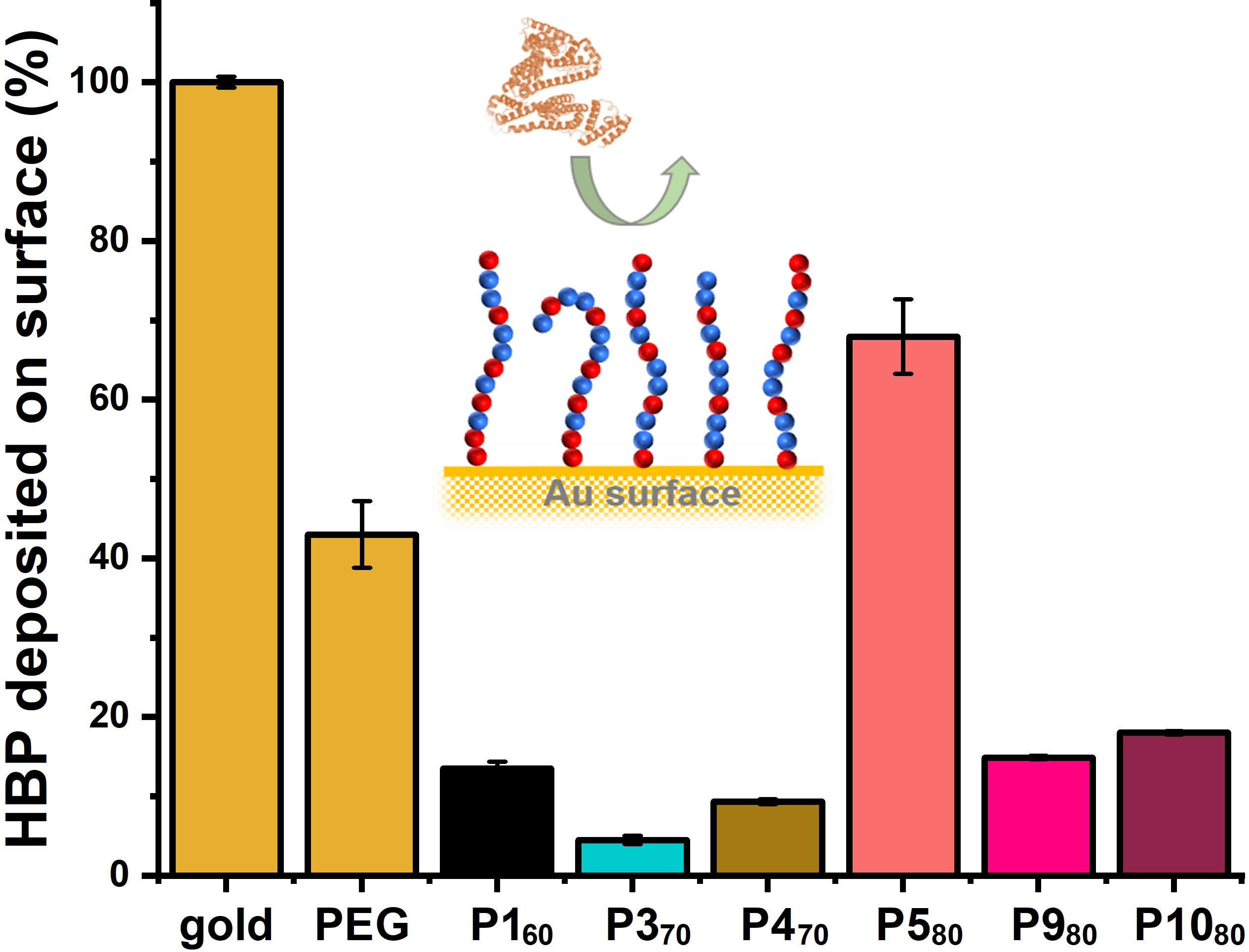


**Figure S19:** Antifouling properties of gold surfaces coated with selected fluorinated copolymers and the reference PEG against whole blood plasma referenced to the non-coated gold surface.


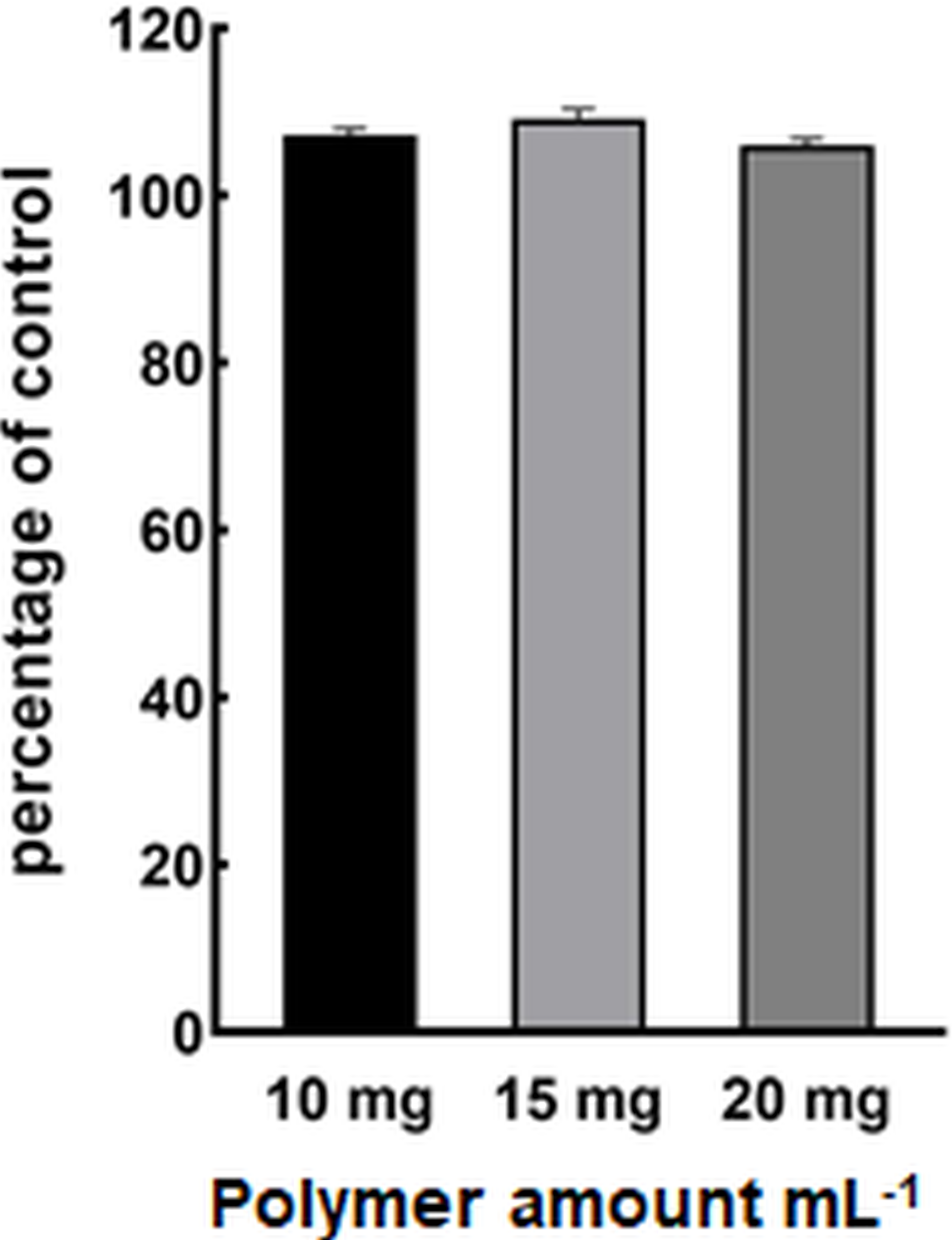


**Figure S20.** 4T1 cell viability expressed as percentage of control. Once the cells reached 70% confluence, the polymer was diluted in fresh growth medium to final concentrations of *c*_pol_ = 10, 15 and 20 mg mL^-1^ and added to the wells in triplicates (100 μL per well). The control cells received fresh growth medium without the polymer. The polymer effect on cell viability was assessed after 24 hours of incubation, followed by 4 hours of incubation with 10% AlamarBlue.


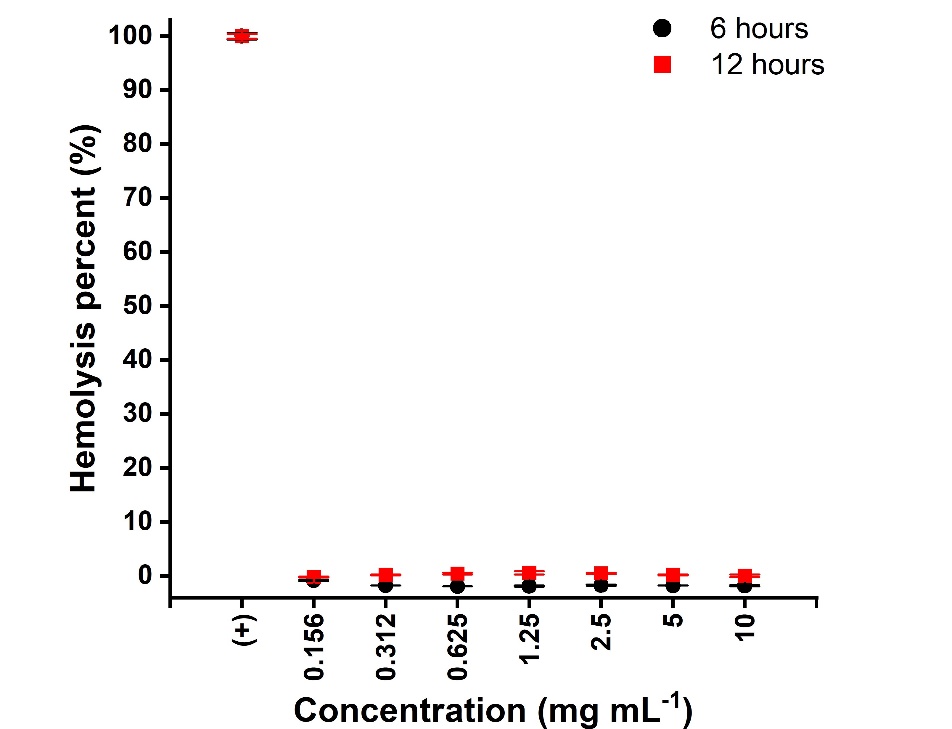


**Figure S21.** Blood compatibility test of P1_60_ expressed as hemolysis percent of positive control showed that the polymer is nonhemolytic. 0.3 mL of diluted RBCs were added to 1 mL given concentration of polymer. As a positive control (+) deionized water was used. According to the ASTM F756-08 standard, hemoglobin release up to 2% was considered as nonhemolytic [4].


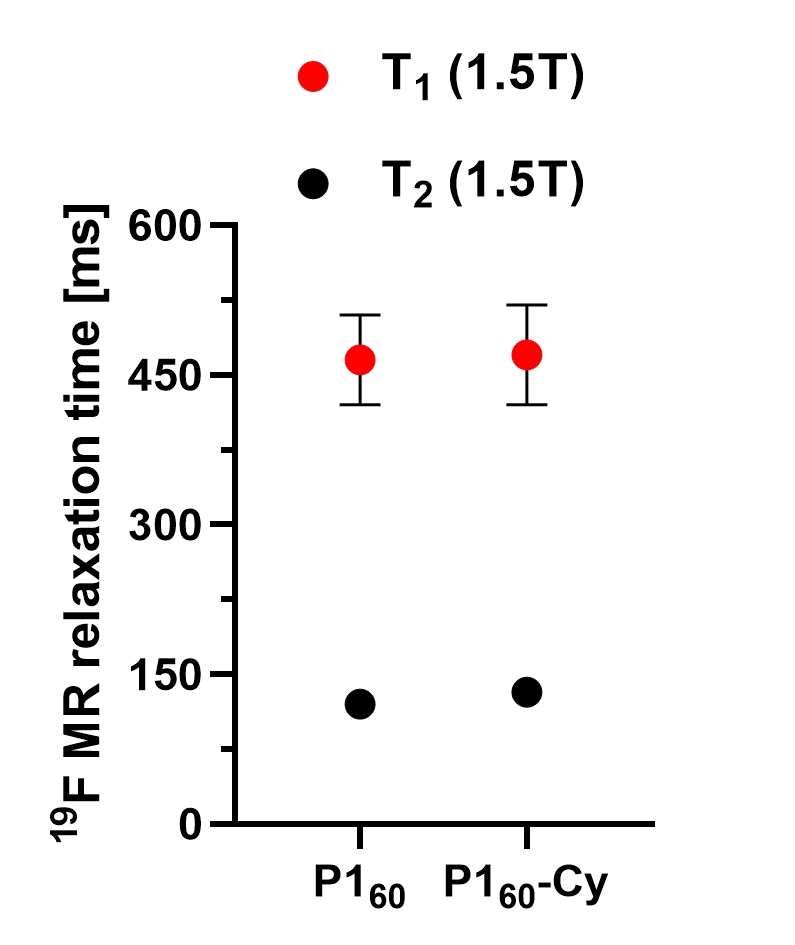


**Figure S22.** Comparison of ^19^F relaxation times of P1_60_ and its fluorescently-labeled high molar mass P1_60_-Cy in water; displaying no distinct difference.


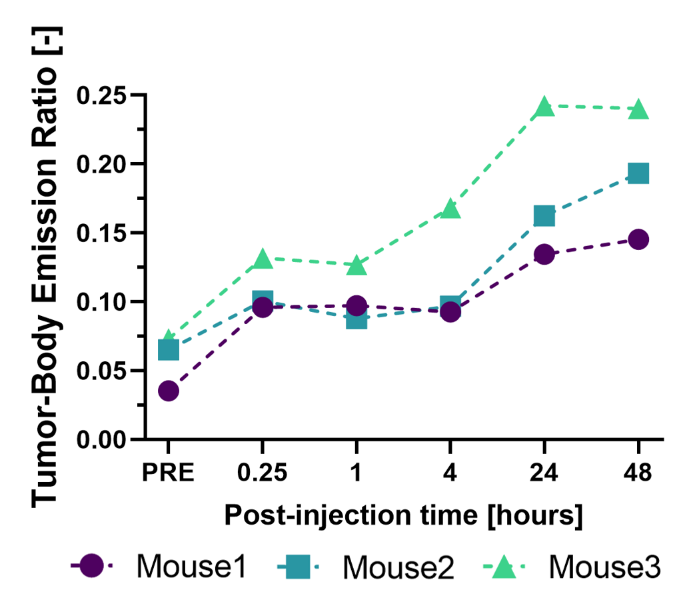


**Figure S23.** Increasing the tumor-to-body ratio of total emission results in P1_60_ tracer accumulation within 4T1 tumors in mice.

**
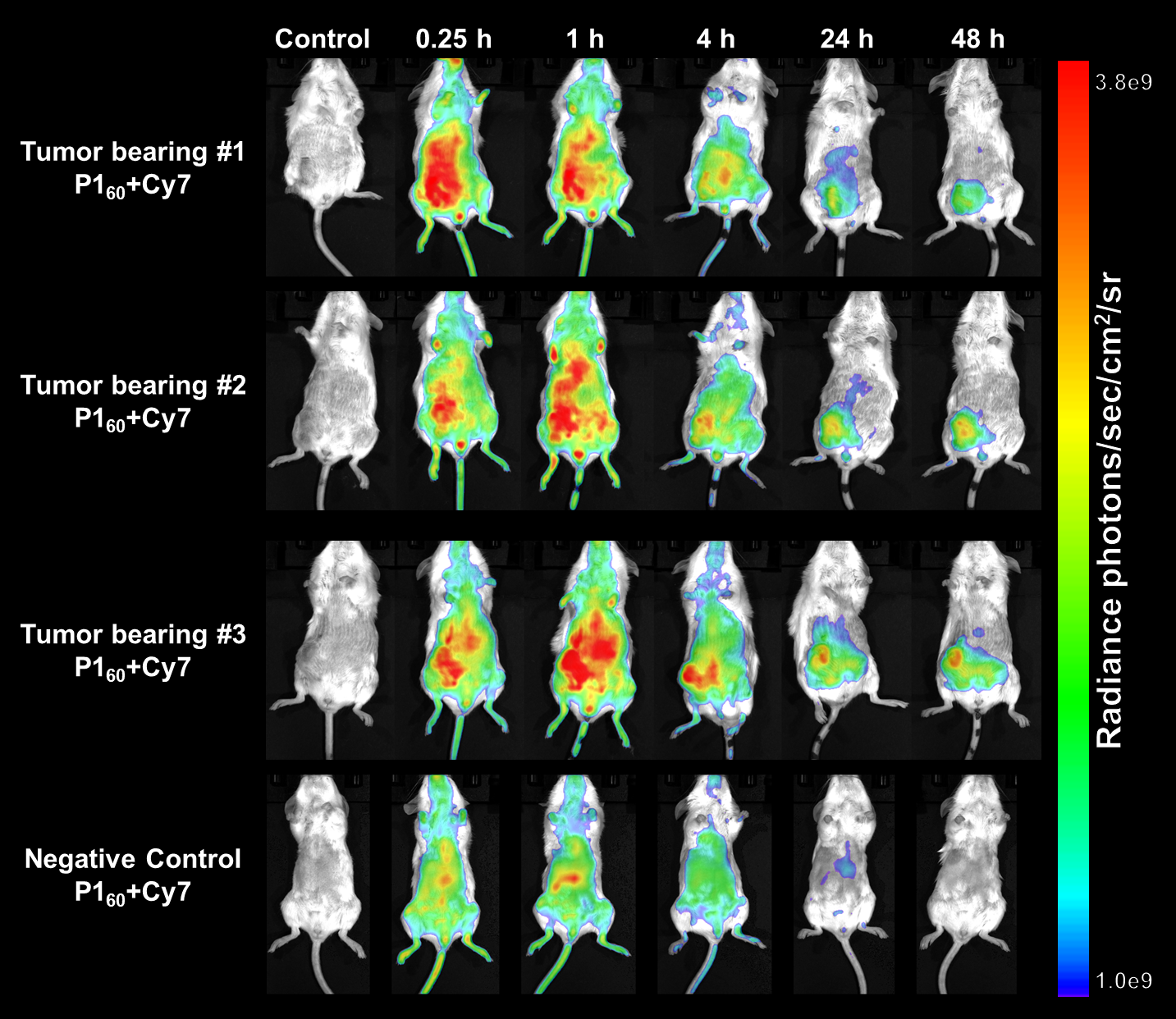
**

**Figure S24.** *In vivo* fluorescence imaging with P1_60_-Cy7 tracer injected into 4T1 tumor-bearing mice and healthy negative control at various time-points after injection.


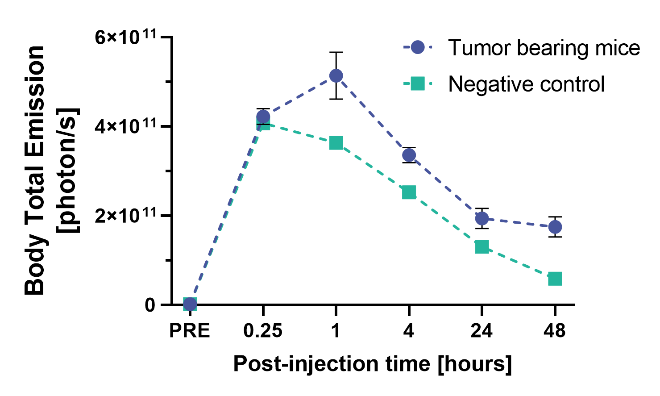


**Figure S25.** Fluorescence imaging of body total emission using P1_60_-Cy7 in 4T1 tumor-bearing mice (blue) and healthy negative control mice (green) before and after intravenous tracer injection reveals polymer accumulation in the tumor of the tumor-bearing mice. In contrast, the body emission in tumor-bearing mice stabilizes over time due to continued tracer accumulation in the tumor, while in healthy control mice, the tracer is gradually eliminated from the body.


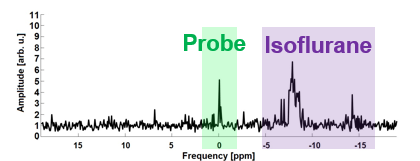


**Figure S26.** Localized in vivo ^19^F MRS signal originating from the tumorous tissue, showcasing distinct peaks that allow for clear separation.


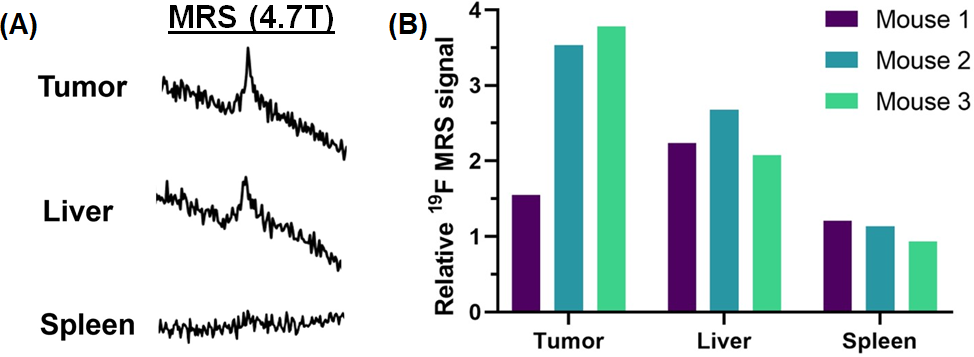


**Figure S27.** Distribution of the tracer P1_60_-Cy7 assessed by (A) *ex vivo* ^19^F MRS in the tumor, liver and spleen (scan time = 30 min) (B) integral of the signal-to-noise ratio. All organs and tumors were extracted 48 hours PI.

**
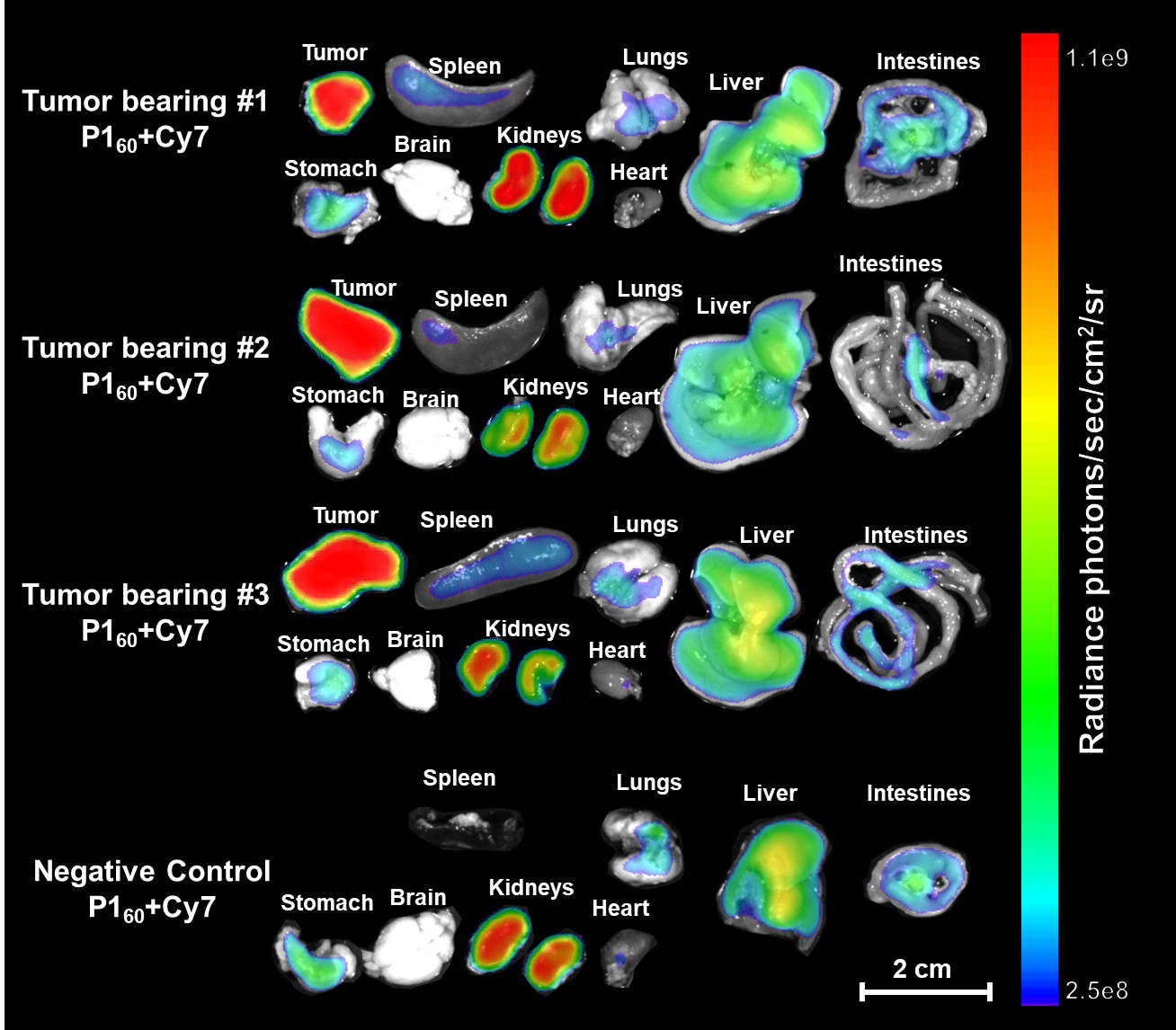
**

**Figure S28.** *Ex vivo* fluorescence imaging with P1_60_-Cy7 tracer injected into 4T1 tumor-bearing mice and healthy negative control; the vital organs and tumor tissue were harvested 48 h after injection.

**
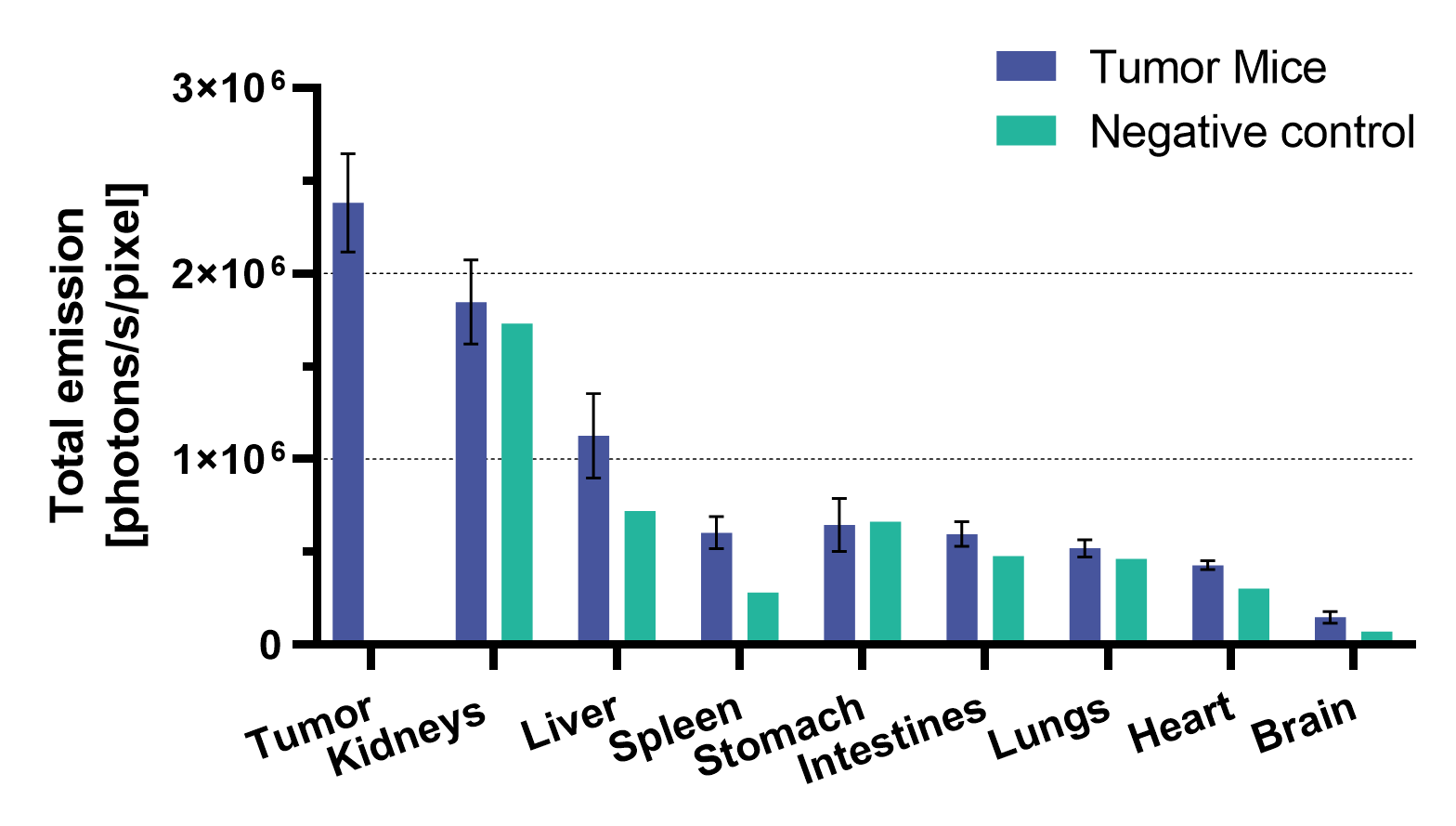
**

**Figure S29.** Ex vivo fluorescence imaging with normalized total emission from vital organs and tumor was performed 48 hours PI. Both the tumor-bearing group and the negative control mouse exhibited a similar biodistribution pattern across all vital organs.

**References**

[1] O. Pop-Georgievski, D. Verreault, M.-O. Diesner, V. Proks, S. Heissler, F. Rypáček, P. Koelsch, *Langmuir* **2012**, *28*, 14273.

[2] Y.-M. Wang, A. Kálosi, Y. Halahovets, I. Romanenko, J. Slabý, J. Homola, J. Svoboda, A. de los S. Pereira, O. Pop-Georgievski, *Polym. Chem.* **2022**, *13*, 3815.

[3] J. Svoboda, N. Lusiani, R. Sivkova, O. Pop-Georgievski, O. Sedlacek, *Macromol. Rapid Commun.* **2023**, *44*, 2300168.

[4] J. Trousil, Z. Syrová, N.-J. K. Dal, D. Rak, R. Konefał, E. Pavlova, J. Matějková, D. Cmarko, P. Kubíčková, O. Pavliš, et al., *Biomacromolecules* **2019**, *20*, 1798.
